# Loss of oocyte Rps26 in mice arrests oocyte growth and causes premature ovarian failure

**DOI:** 10.1038/s41419-018-1196-3

**Published:** 2018-11-19

**Authors:** Xiao-Man Liu, Ming-Qi Yan, Shu-Yan Ji, Qian-Qian Sha, Tao Huang, Han Zhao, Hong-Bin Liu, Heng-Yu Fan, Zi-Jiang Chen

**Affiliations:** 1 Center for Reproductive Medicine, Shandong Provincial Hospital Affiliated to Shandong University, Jinan, 250001 China; 2 National Research Center for Assisted Reproductive Technology and Reproductive Genetics, Jinan, 250001 China; 3 The Key Laboratory for Reproductive Endocrinology, Shandong University, Ministry of Education, Jinan, 250001 China; 4 Life Sciences Institute and Innovation Center for Cell Signaling Network, Zhejiang University, Hangzhou, 310058 China; 5 Center for Reproductive Medicine, Ren Ji Hospital, School of Medicine, Shanghai Jiao Tong University, Shanghai, 200135 China; 6 Shanghai Key Laboratory for Assisted Reproduction and Reproductive Genetics, Shanghai, 200135 China

## Abstract

Global transcriptional activity increases as oocytes grow and is silenced in fully grown oocytes. Thus, the chromatin configuration varies during oocyte growth, but the molecular mechanisms regulating these changes remain to be clarified. Here, we studied a susceptibility gene of polycystic ovary syndrome (PCOS), *RPS26*, which is a ribosomal protein-encoding gene that is highly expressed in the ovary, but the functions of which remain unknown. Specific knockout of *Rps26* in mouse oocytes resulted in retarded follicle development from pre-antral follicles to antral follicles, while the chromatin configurations of the oocytes were arrested at the transition from the non-surrounded nucleolus (NSN) to surrounded nucleolus (SN)-type. As a consequence, all oocytes died by postnatal day 84 resulting in premature ovarian failure (POF). Loss of *Rps26* in oocytes led to decreased mRNA transcription and low levels of histone trimethylation on H3K4/H3K9 and DNA methylation at 5-cytosine, high levels of which are required for oocytes to transform from NSN to SN-type. Low protein levels of oocyte-derived growth differentiation factor 9, bone morphogenetic protein 15, and the oocyte-granulosa cell gap junction protein connexin 37 inhibited oocyte growth and retarded follicle development. The disruption of the phosphoinositide 3-kinase/protein kinase B/Forkhead box O-3a pathway contributed to oocyte death and follicle atresia. These results provide genetic clues for the clinical diagnosis of POF, especially in PCOS patients without treatment.

## Introduction

Ribosomal proteins play extra-ribosomal roles in cell proliferation, cell cycle progression, and RNA transcription^1–3^. RPS26 is a highly conserved ribosomal protein with about 86.5% similarity in eukaryotes. It has a potent mRNA-binding domain in the 62-YxxPKxYxK-70 amino acid sequence (Fig. S1A)^4^, which is suggested to be involved in the translation of a subset of mRNAs^5^. *RPS26* is reported to be highly expressed in human ovary, and has been implicated in the polycystic ovary syndrome (PCOS)^6,7^, which suggest a potential role in female reproduction.

Follicle maturation and oocyte quality are important for ovarian functions in female reproduction^8–10^. Mammalian follicle maturation is tightly connected with the growth of oocyte, and the oocytes control communications between themself and their adjacent granulosa cells^11,12^. Since *RPS26* is implicated in the ovarian functions, we were interested in evaluating the regulatory role of *RPS26* during oocyte growth and the in vivo functions in ovarian follicle development.

The chromatin configuration in oocytes changes during oocyte growth, and gene expression is globally silenced in fully grown germinal vesicle (GV) oocytes^13^. There are two types of GV oocytes, the non-surrounded nucleolus (NSN) and the surrounded nucleolus (SN), these types of oocytes have different chromatin configurations and transcriptional activities^14,15^. In SN-type oocytes, the chromatin is highly condensed and gathered around the nucleolus, and the gene transcription is globally silenced. In NSN-type oocytes, the chromatin is relaxed and does not surround the nucleolus, and the gene transcription is globally active^13,16^. Chromatin in fully grown oocytes generally has SN-type configuration, while most growing oocytes show NSN-type configuration^17^. The regulatory mechanism involved in the transition from NSN to SN oocyte remains to be studied.

Chromatin configuration is associated with epigenetic modifications that play important roles in gene expression, such as histone and DNA methylation^13,18^. Methylation of histone 3 at lysine 4 (H3K4) and lysine 9 (H3K9) are highly conserved epigenetic markers, and these are associated with active transcription and gene silencing^19^. In mammals, the level of DNA methylation is dependent on the histone methylation on specific genomic sites, and DNA methylation regulates gene expression according to the amount of methylation^20^. Ribosomal genes are located at the outer periphery of the nucleolus in NSN-type oocytes, and RNA polymerase-I dependent transcription correlates with the specific ultrastructure of nucleolus^17^. However, whether these ribosomal genes are involved in changes to the chromatin configuration or RNA transcription remains unclear.

In ovary, oocyte growth is of great importance for the proliferation and metabolism of granulosa cells as well as for the development of follicles. The phosphoinositide 3-kinase (PI3K)/protein kinase B (AKT)/Forkhead box O-3a (FOXO3a) pathway regulates primordial follicle activation and production of mature oocytes^21–23^. Cell survival and proliferation are promoted when AKT is phosphorylated at Ser473, or the substrates of AKT such as the Forkhead transcription factors are inactivated^24^. In particular, FOXO3a can be directly phosphorylated by AKT, and phosphorylation of the Ser253 residue is crucial for shuttling FOXO3a from nucleus to cytoplasm. FOXO3a is one of the transcription factors that are essential for maintaining the dormant state of primordial follicles in the ovary^25^. Constitutively active FOXO3a in oocytes leads to retarded oocyte growth and follicular development, while lack of FOXO3a leads to global follicle activation and subsequent premature ovarian failure (POF)^26,27^.

Oocytes control the bidirectional communication between oocytes and the connected granulosa cells, which is required for oocyte growth and follicle development. Growth differentiation factor 9 (GDF9) and bone morphogenetic proteins 15 (BMP15) are oocyte-derived growth and differentiation secretory factors that are regulators of folliculogenesis and granulosa cell differentiation^28,29^. Gap junctions are bridges between oocytes and their adjacent granulosa cells, which are crucial for the secretion of GDF9 and BMP15 into the adjacent granulosa cells, and for the diffusion of cAMP/cGMP from granulosa cells to oocyte^30,31^. Connexin 37 (CX37) is a gap junction protein localized at the oocyte surface and exclusively connects the adjacent granulosa cells, loss of CX37 leads to stop of oocyte growth and loss of meiotic competence^32,33^.

In this work, we constructed oocyte-specific *Rps26* knockout mouse model, and elucidated the functions of *Rps26* in oocyte growth and follicle development.

## Results

### Oocyte Rps26 is required for female fertility

**Fig. 1:**
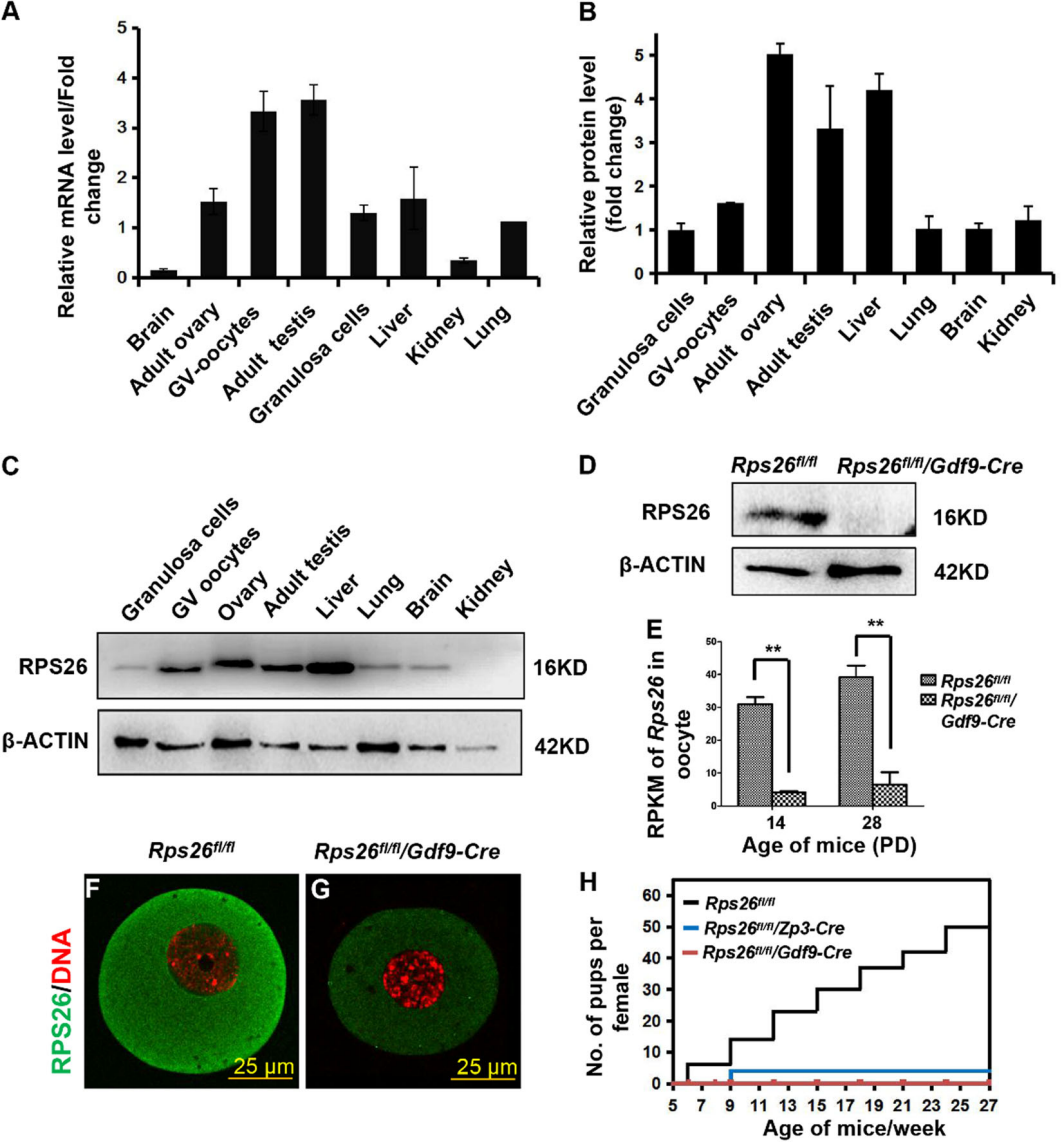
*Rps26* is highly expressed in ovarian oocytes and is indispensable for female fertility. **a** Real-time PCR analyses showing relatively high levels of *Rps26* mRNA in GV oocytes and adult testis. The *Rps26* mRNA levels of all samples were compared to the *Rps26* mRNA level in the lung. **b** Western blot analyses showing relatively high expressions of Rps26 protein in the adult ovary, testis, and liver. The Rps26 protein levels of all samples were compared to the Rps26 protein level in granulosa cells. Protein levels were quantified with the Image J software, and the average value of each sample was calculated using Microsoft Office Excel. **c** Representative images of the western blot analysis from **b** showing the expression of the Rps26 protein in different tissues and organs. **d** Western blot analysis showing that expression of Rps26 protein in GV oocytes from the ovaries of *Rps26*^*fl/fl*^*/Gdf9-Cre* mice was undetectable compared with that in *Rps26*^*fl/fl*^ mice. **e** mRNA sequencing analysis showing significantly decreased *Rps26* mRNA expression in the oocytes of *Rps26*^*fl/fl*^*/Gdf9-Cre* mouse ovaries at PD14 and PD28. **p *<* 0.01 as calculated by two-tailed Student’s *t*-tests. **f**, **g** Immunofluorescence results for the expression of Rps26 in oocytes collected from *Rps26*^*fl/fl*^ mice (**f**) and *Rps26*^*fl/fl*^*/Gdf9-Cre* mice (**g**). **h** Cumulative numbers of pups per female of the indicated genotypes. *Rps26*^*fl/fl*^*/Gdf9-Cre* females were infertile

The mRNA and protein expression of Rps26 was high in the ovarian GV oocytes and granulosa cells compared to other tissues as analyzed with real-time reverse transcription polymerase chain reaction (RT-PCR) (Fig. 1a) and western blot assays (Fig. 1b, c). Rps26 has a potent mRNA-binding domain in the 62-YxxPKxYxK-70 amino acid sequence (Fig. S1A)^4^. To study the *in vivo* function of *Rps26* in female reproduction, we targeted the mRNA-binding domain and generated *Rps26* floxed (*Rps26*^*fl/fl*^) mice, and crossed them with *Gdf9-Cre* or *Zp3-Cre* mice (these Cre DNA recombinases are specifically expressed in oocytes at postnatal day (PD) 3 and PD5, respectively^34–36^) to obtain mice with oocyte-specific deletion of *Rps26* (Fig. S1C). The Rps26 protein was almost undetectable in the oocytes of *Rps26*^*fl/fl*^*/Gdf9-Cre* mice at PD14, as validated with western blot analysis (Fig. 1d), RNA sequencing (Fig. 1e), and immunofluorescence (Fig. 1f, g). After crosses with male *Rps26*^*fl/fl*^ mice, female *Rps26*^*fl/fl*^*/Gdf9-Cre* and *Rps26*^*fl/fl*^*/Zp3-Cre* mice were found infertile (Fig. 1h).

## Rps26 is essential for the follicle development and oocyte maturation

**Fig. 2:**
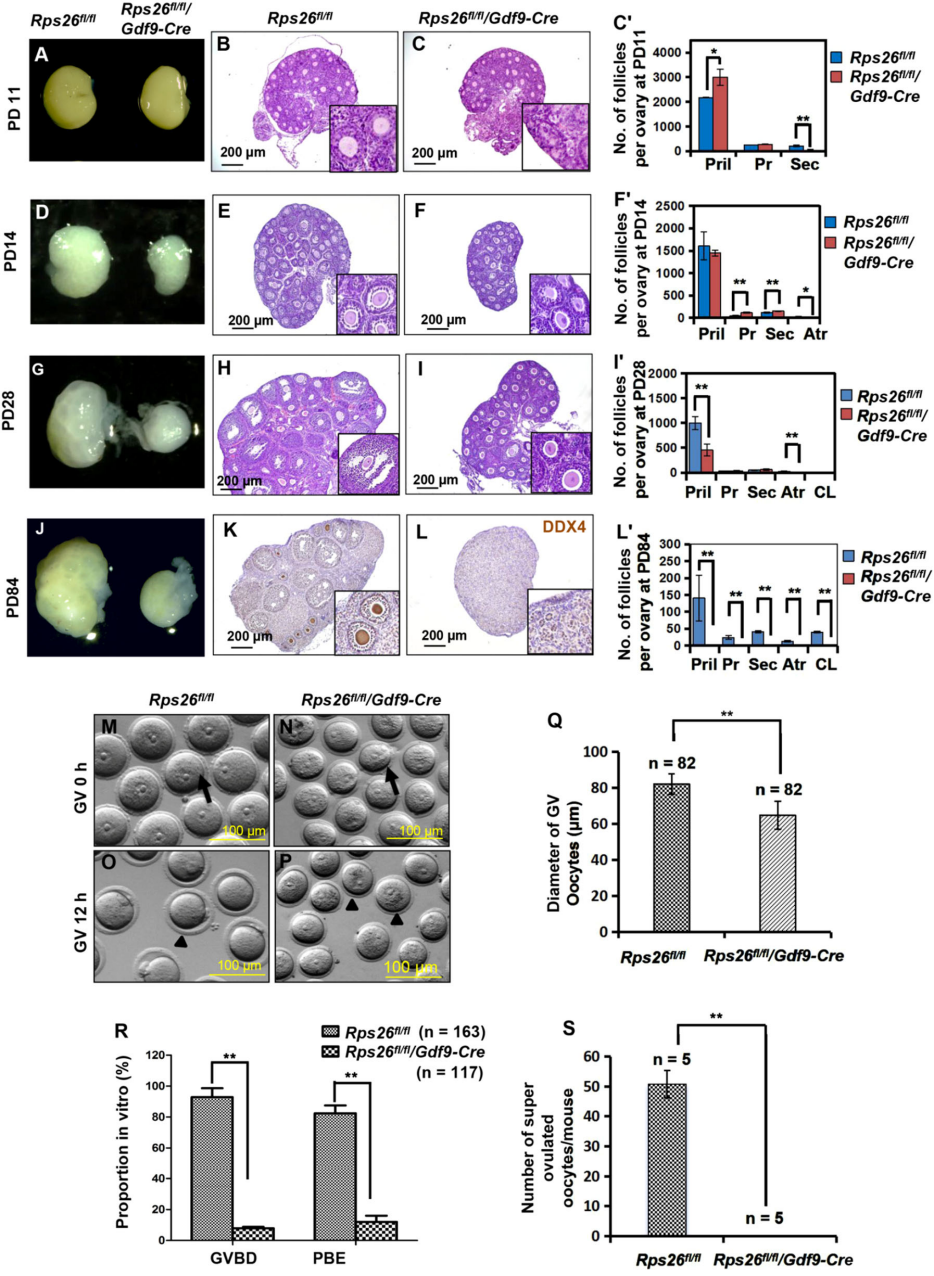
Rps26 is essential for follicle development and oocyte growth. **a** Ovaries of PD11 *Rps26*^*fl/fl*^*/Gdf9-Cre* mice and *Rps26*^*fl/fl*^ mice were similar in size. **b**, **c** Hematoxylin/eosin staining of the paraffin slides of ovaries showing the similar morphologies of the ovaries at PD11 from *Rps26*^*fl/fl*^ mice (**b**) and *Rps26*^*fl/fl*^*/Gdf9-Cre* mice (**c**). **c**’ Statistical analysis of the numbers of follicles in the ovaries of *Rps26*^*fl/fl*^ mice (**b**) and *Rps26*^*fl/fl*^*/Gdf9-Cre* mice (**c**). *p *<* 0.05, **p *<* 0.01 as calculated by two-tailed Student’s *t*-tests. **d** Ovaries of *Rps26*^*fl/fl*^*/Gdf9-Cre* mice at PD14 were smaller than those of *Rps26*^*fl/fl*^ mice. **e**, **f** The ovarian morphology at PD14 showing that there was arrest at the pre-antral follicle stage in the ovaries of *Rps26*^*fl/fl*^*/Gdf9-Cre* mice (**f**, insert) compared to *Rps26*^*fl/fl*^ mice (**e**, insert). **f**’ Statistical analysis of the numbers of follicles in the ovaries of *Rps26*^*fl/fl*^ mice (**e**) and *Rps26*^*fl/fl*^*/Gdf9-Cre* mice (**f**). *p *<* 0.05, **p *<* 0.01 as calculated by two-tailed Student’s *t*-tests. **g**, **j** Ovaries of *Rps26*^*fl/fl*^*/Gdf9-Cre* mice at PD28 (**g**) and PD84 (**j**) were smaller compared to ovaries of *Rps26*^*fl/fl*^ mice. **h**, **i** Ovarian morphology in *Rps26*^*fl/fl*^*/Gdf9-Cre* mice at PD28 also showing arrest at the pre-antral follicle stage (**i**, insert) compared to the ovarian follicles in *Rps26*^*fl/fl*^ mice that had developed into pre-ovulatory and antral follicles (**h**, insert). **i**’ Statistical analysis of the numbers of follicles in the ovaries of *Rps26*^*fl/fl*^ mice (**h**) and *Rps26*^*fl/fl*^*/Gdf9-Cre* mice (**i**). **p *<* 0.01 as calculated by two-tailed Student’s *t*-tests. **k**, **l** Immunohistochemistry for the oocyte marker DEAD-Box Helicase 4 (DDX4) on ovarian slides of PD84 *Rps26*^*fl/fl*^*/Gdf9-Cre* mice (**l**, insert) showing no oocytes compared to those of *Rps26*^*fl/fl*^ mice (**k**, insert). **l**’ Statistical analysis of the numbers of follicles in the ovaries of *Rps26*^*fl/fl*^ mice (**k**) and *Rps26*^*fl/fl*^*/Gdf9-Cre* mice (**l**). **p *<* 0.01 as calculated by two-tailed Student’s *t*-tests. **m**, **n** GV oocytes collected from the ovaries of *Rps26*^*fl/fl*^ mice (**m**) were larger in diameter than those from the ovaries of *Rps26*^*fl/fl*^*/Gdf9-Cre* mice (**n**) at PD21–28. **o**, **p** After 12 h in vitro culture, oocyte meiosis was arrested at the GV stage in *Rps26*^*fl/fl*^*/Gdf9-Cre* mice (**p**), while the oocytes of *Rps26*^*fl/fl*^ mice had developed into MII oocytes (**o**). **q** The quantification of the diameters of GV oocytes shown in m and n showing that oocytes collected from the ovaries of *Rps26*^*fl/fl*^*/Gdf9-Cre* mice were significantly smaller than GV oocytes from the ovaries of *Rps26*^*fl/fl*^ mice. **p *<* 0.01 as calculated by two-tailed Student’s *t*-tests. **r** Quantification of GVBD and PBE (shown in **o** and **p**) oocytes during meiosis. The proportions of GVBD and PBE were significantly decreased in vitro in the oocytes from the ovaries of *Rps26*^*fl/fl*^*/Gdf9-Cre* mice compared to those from *Rps26*^*fl/fl*^ mice. **p *<* 0.01 as calculated by two-tailed Student’s *t*-tests. **s** Oocyte superovulation was inhibited in *Rps26*^*fl/fl*^*/Gdf9-Cre* mice compared to *Rps26*^*fl/fl*^ mice. ** *p* *<* 0.01 as calculated by two-tailed Student’s *t*-tests

To clarify the infertility of *Rps26*^*fl/fl*^*/Gdf9-Cre* female mice, we observed the morphology of ovaries at different ages and analyzed the composition of follicles according to the classification of primordial, primary, secondary (pre-antral), antral follicles and corpus luteum (Fig. S2A). The total number of follicles in *Rps26*^*fl/fl*^*/Gdf9-Cre* ovaries was significantly increased compared to *Rps26*^*fl/fl*^ mice at PD11, but gradually declined from PD14 to PD84, and the numbers of follicles at PD28, PD42, and PD84 were significantly lower compared to *Rps26*^*fl/fl*^ mice (Fig. S2B). *Rps26*^*fl/fl*^*/Gdf9-Cre* ovaries showed no difference in size at PD11 (Fig. 2a), but contained significantly more primordial follicles and fewer secondary follicles compared to *Rps26*^*fl/fl*^ ovaries (Fig. 2b, c, c’), which indicated that most of the follicles had been arrested at primary follicle stage in *Rps26*^*fl/fl*^*/Gdf9-Cre* ovaries. From PD14 to PD84, the *Rps26*^*fl/fl*^*/Gdf9-Cre* ovaries were smaller in size compared to *Rps26*^*fl/fl*^ ovaries (Fig. 2d, g, j). At PD14, most follicles were at the primordial stage, but some had advanced to the antral stage in *Rps26*^*fl/fl*^ ovaries (Fig. 2e, insert), while no antral follicles were seen in *Rps26*^*fl/fl*^*/Gdf9-Cre* ovaries, although more pre-antral follicles were observed than that in *Rps26*^*fl/fl*^ (Fig. 2f, insert, **f**’). At PD28, most follicles in *Rps26*^*fl/fl*^*/Gdf9-Cre* ovaries were still arrested at the pre-antral follicular stage compared to *Rps26*^*fl/fl*^ ovaries (Fig. 2h, i, inserts, **i**’), suggesting that follicle developmental arrest had occurred at the pre-antral follicle to antral follicle stage in *Rps26*^*fl/fl*^*/Gdf9-Cre* ovaries by PD28. However, it must be noted that a few follicles in *Rps26*^*fl/fl*^*/Gdf9-Cre* ovary could still develop into antral follicles. At PD21, the number of primary and secondary follicles had increased significantly in *Rps26*^*fl/fl*^*/Gdf9-Cre* ovaries, while few antral follicles were detected (Fig. S2C), although occasional development of follicles into antral follicles was observed (Fig. S2F, arrow). The *Rps26*^*fl/fl*^*/Gdf9-Cre* ovary showed a very poor response to the treatment with pregnant mare serum gonadotropin (PMSG) 44 h, indicating that the follicles, excluding the ovulated follicles (Fig. S2H, arrow), were mostly arrested in the pre-antral stage (Fig. S2H, insert) compared with *Rps26*^*fl/fl*^ ovaries (Fig. S2G, arrow). At PD42, the numbers of follicles, including the pre-antral and antral follicles, were reduced due to follicle atresia (Fig. S2D, J, insert), and only a few follicles developed into antral follicles and formed corpora lutea after ovulation in *Rps26*^*fl/fl*^*/Gdf9-Cre* ovary compared to *Rps26*^*fl/fl*^ ovary (Fig. S2I, J, arrows, inserts). As a result, all follicles disappeared by PD84, and no oocytes were observed in *Rps26*^*fl/fl*^*/Gdf9-Cre* ovary (Fig. 2l, insert, **l**’). The phenotype was compromised in *Rps26*^*fl/fl*^*/Zp3-Cre* mice, some primary follicles were observed at PD84 (Fig. S3I), while the mice were still infertile. Morphologies of the ovaries suggested that *Rps26*^*fl/fl*^*/Gdf9-Cre* ovaries had lost all the oocytes and follicles, but how the oocytes died out remained unknown.

GV oocytes from PD21 mice were dramatically smaller after the deletion of *Rps26* by Gdf9-Cre (Fig. 2n, arrow) or Zp3-Cre (Fig. S3B, arrow) as compared with *Rps26*^*fl/fl*^ mice (Fig. 2m, arrow; Fig. S3A, arrow). The average diameter was about 80 μm for oocytes from *Rps26*^*fl/fl*^ mice, but only about 65 μm for oocytes from *Rps26*^*fl/fl*^*/Gdf9-Cre* mice (Fig. 2q), and about 69 μm for oocytes from *Rps26*^*fl/fl*^*/Zp3-Cre* mice (Fig. S3E). Following 12 h of in vitro culture, about 80% of the oocytes had extruded the first polar body in the *Rps26*^*fl/fl*^ mice (Fig. 2o arrowhead, R; Fig. S3C arrowhead, S3F), but about 90% of *Rps26*^*fl/fl*^*/Gdf9-Cre* oocytes (Fig. 2p arrowhead, **r**) and about 85.5% of *Rps26*^*fl/fl*^*/Zp3-Cre* oocytes (Fig. S3D arrowhead, S3F) were arrested at the GV stage. Very few oocytes underwent ovulation due to poor response to the administration of PMSG (Fig. 2s and Fig. S3G).

These results suggested that oocytes of *Rps26*^*fl/fl*^*/Gdf9-Cre* mice at PD21–28 were mostly arrested in pre-antral follicular stage and lacked the competence to undergo meiotic maturation.

### Rps26 might regulate chromatin configuration by reducing the trimethylation level at H3K4 and H3K9 in growing oocytes

**Fig. 3:**
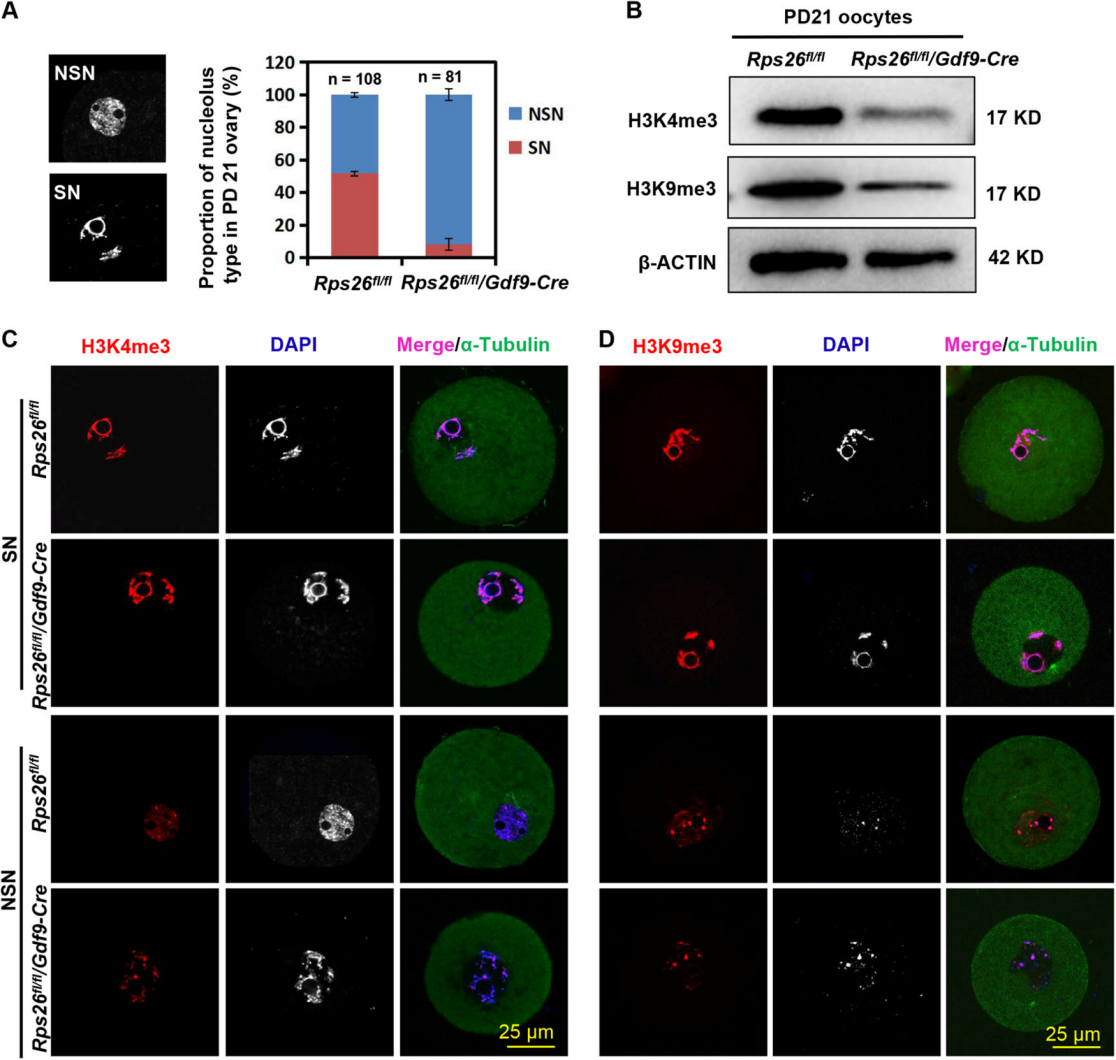
Rps26 regulated oocyte chromatin configuration as mediated by trimethylation of H3K4 and H3K9. **a** The proportion of the indicated NSN and SN nucleolus types in PD21 ovaries showing that most of the oocytes in *Rps26*^*fl/fl*^*/Gdf9-Cre* mice were NSN type. **b** Protein levels of the trimethylation of H3K4 (H3K4me3) and H3K9 (H3K9me3) were significantly decreased in the GV oocytes of the *Rps26*^*fl/fl*^*/Gdf9-Cre* mice compared to the levels in *Rps26*^*fl/fl*^ mice. **c**–**d** Immunofluorescence of H3K4me3 (**c**) and H3K9me3 (**d**) in PD21 GV oocytes (SN and NSN types) showing that low levels of H3K4me3 and H3K9me3 in *Rps26*^*fl/fl*^*/Gdf9-Cre* mice correlated with the high proportion of NSN type oocytes

During growth process, oocytes show an increase in epigenetic modifications, including histone and DNA modifications, they gradually acquire the developmental potency and competence for meiotic maturation. At PD21, more than 50% of the oocytes were SN-type in *Rps26*^*fl/fl*^ mice, while about 90% of the oocytes were NSN-type in *Rps26*^*fl/fl*^*/Gdf9-Cre* mice (Fig. 3a). This suggests the failure of the NSN to SN transition in *Rps26*^*fl/fl*^*/Gdf9-Cre* oocytes. H3K4 trimethylation (H3K4me3) levels were high in SN-type oocytes and low in NSN-type oocytes in *Rps26*^*fl/fl*^ mice (Fig. 3c). Significantly low levels of H3K4me3 were detected in the oocytes from *Rps26*^*fl/fl*^*/Gdf9-Cre* mice (Fig. 3b), and the level of H3K4me3 seemed to be related with the nucleolus types (Fig. 3c). This was consistent with the observation that most oocytes in *Rps26*^*fl/fl*^*/Gdf9-Cre* mice were NSN-type (Fig. 3a). H3K9 trimethylation (H3K9me3) was observed at high levels in the SN-type oocytes of both *Rps26*^*fl/fl*^ mice and *Rps26*^*fl/fl*^*/Gdf9-Cre* mice, but at low levels in the NSN-type oocytes of both *Rps26*^*fl/fl*^ and *Rps26*^*fl/fl*^*/Gdf9-Cre* mice (Fig. 3b, d). The methylation of DNA at the fifth cytosine (5-methylcytosine, 5mC) is a conserved epigenetic marker that is involved in the regulation of gene expression. The 5mC level was lower in *Rps26*^*fl/fl*^*/Gdf9-Cre* oocytes compared to *Rps26*^*fl/fl*^ oocytes (Fig. S4). These results indicated that the oocytes of *Rps26*^*fl/fl*^*/Gdf9-Cre* mice suffered from defects in histone and DNA methylations, and these defects might be the cause or the consequence of the failure to undergo NSN to SN transition.

### *Rps26* deletion decreased the mRNA synthetic activity and transcriptional activity in oocytes

**Fig. 4:**
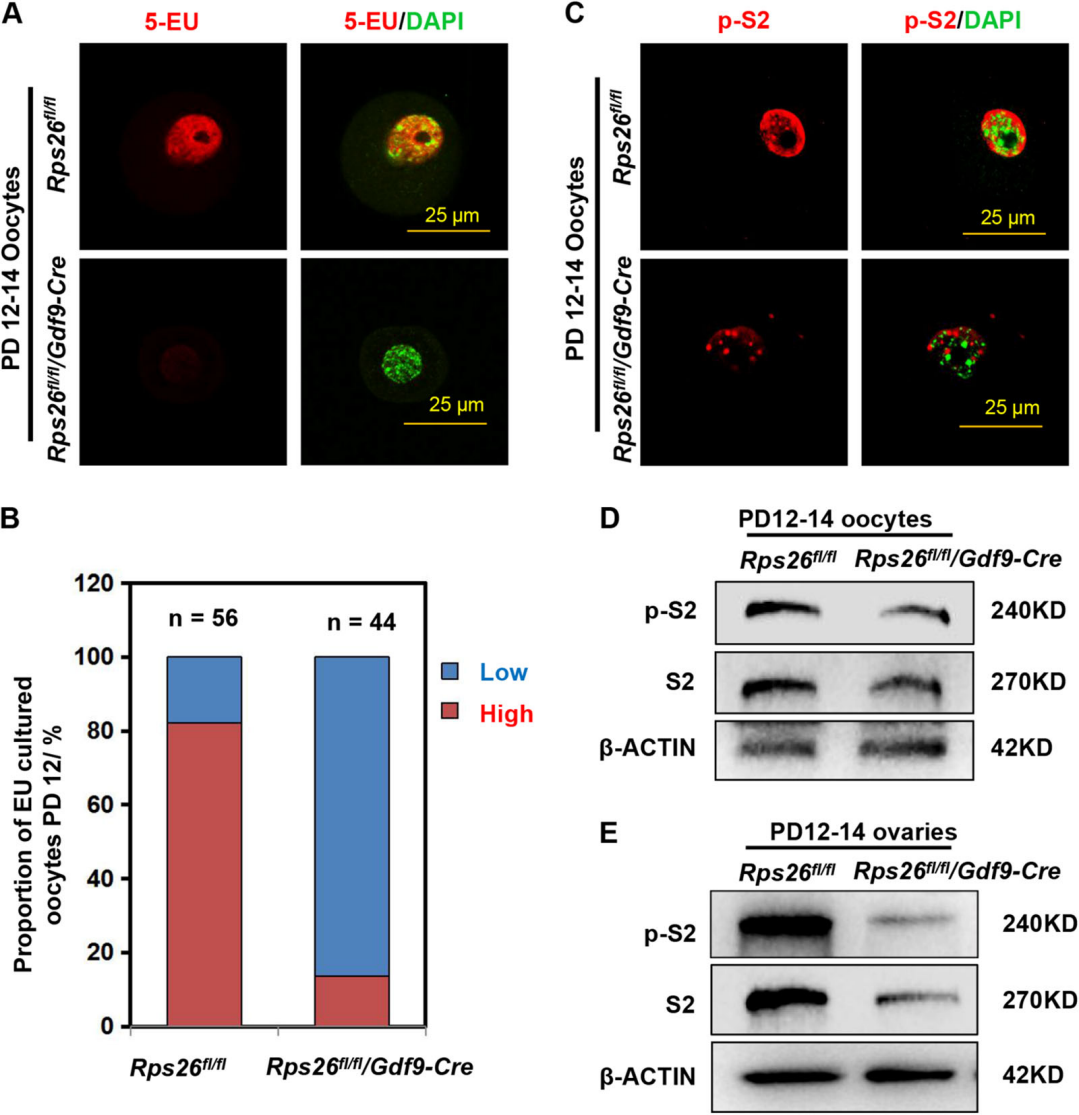
Rps26 is required for RNA synthesis and transcription in growing oocytes. **a** The Click-iT assay of the oocytes from PD12–14 mice showing very low 5-EU signal in oocytes from *Rps26*^*fl/fl*^*/Gdf9-Cre* mice and high 5-EU signal in oocytes from *Rps26*^*fl/fl*^ mice. **b** Quantification of oocytes with high and low 5-EU signals in the indicated genotypes from the assay shown in **a**. **c** Immunofluorescence of p-S2 showing reduced signal in the nuclei of *Rps26*^*fl/fl*^*/Gdf9-Cre* oocytes compared with *Rps26*^*fl/fl*^ oocytes from PD12–14 mice. **d**, **e** Western blot assays showing that protein levels of phospho-RNA polymerase II (p-S2) and total RNA polymerase II (RNA pol II) in oocytes (**d**) and the whole ovaries (**e**) were decreased in *Rps26*^*fl/fl*^*/Gdf9-Cre* mice compared to *Rps26*^*fl/fl*^ mice

The alkyne-modified nucleoside 5-ethynyl uridine (5-EU) was used to label newly synthesized RNA, thus allows temporal detection of global RNA synthesis. 5-EU was globally detected in GV oocytes of *Rps26*^*fl/fl*^ mice at PD12–14; however, about 90% of the *Rps26*^*fl/fl*^*/Gdf9-Cre* oocytes showed low 5-EU signal (Fig. 4a, b). This indicates the RNA synthesis activity was greatly reduced in *Rps26*^*fl/fl*^*/Gdf9-Cre* oocytes. The initiation of RNA transcription is mainly catalyzed by the RNA polymerases, RNA polymerase II is the key enzyme that synthesizes the precursors of most mRNAs. The phosphorylation at the second serine (Ser2) of carboxyl-terminal domain (CTD) of RNA polymerase II (p-S2) is critical for the activation of transcript elongation. We evaluated p-S2 levels by immunofluorescence, low levels of p-S2 in the nuclei was found in *Rps26*^*fl/fl*^*/Gdf9-Cre* oocytes compared to *Rps26*^*fl/fl*^ oocytes from PD12–14 mice (Fig. 4c). Immunohistochemistry results showed that p-S2 was present in the nuclei of both oocytes and granulosa cells in *Rps26*^*fl/fl*^ mice, and low in oocytes but not affected in granulosa cells of *Rps26*^*fl/fl*^*/Gdf9-Cre* mice (Fig. S5). In oocytes and ovaries, protein levels of p-S2 and the total RNA polymerase II were all reduced in *Rps26*^*fl/fl*^*/Gdf9-Cre* mice compared to *Rps26*^*fl/fl*^ mice (Fig. 4d, e). This suggested that the transcriptional activity might be significantly reduced in *Rps26*^*fl/fl*^*/Gdf9-Cre* oocytes.

**Fig. 5:**
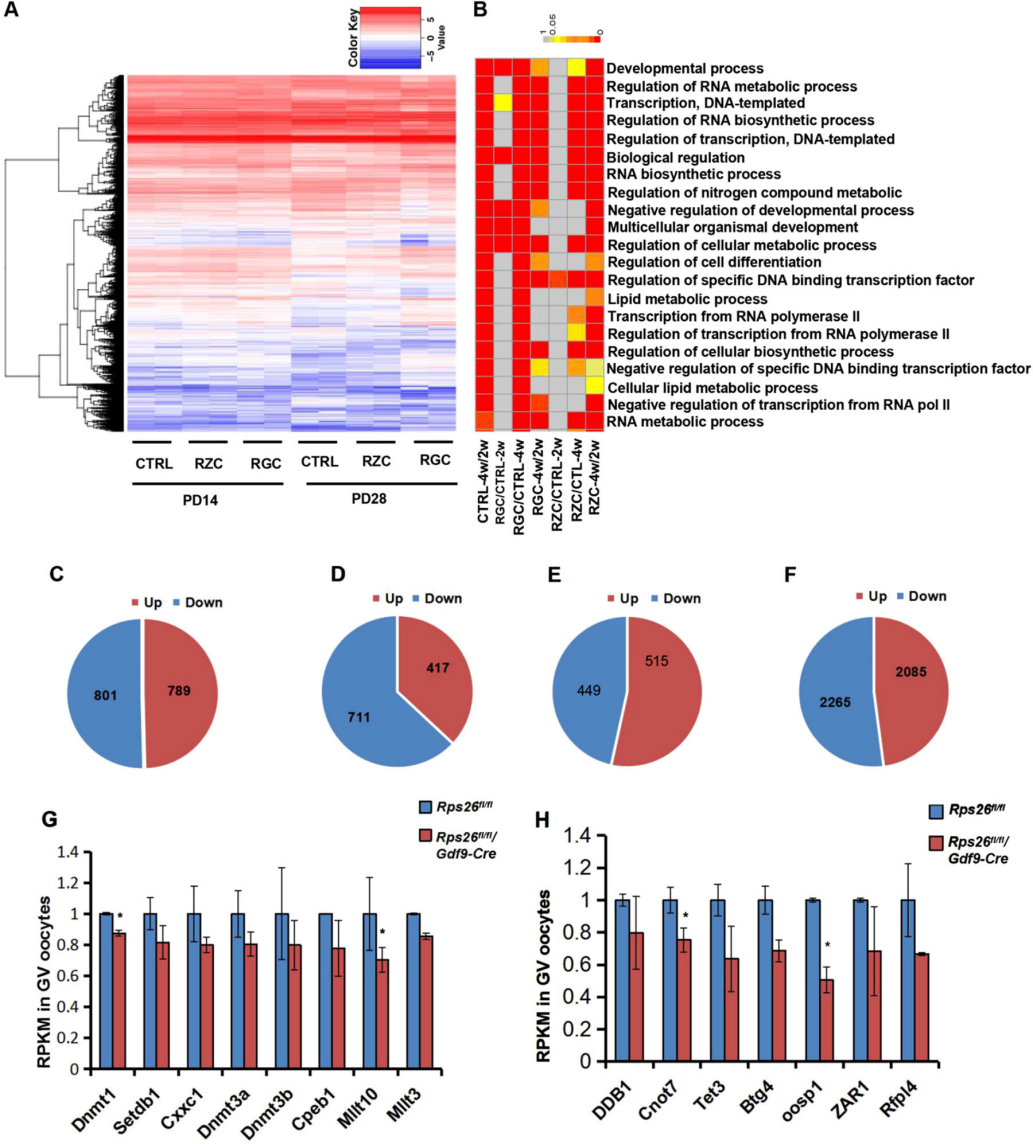
Rps26 regulates the mRNA transcription involved in methylation modifications and developmental processes. **a** Heatmap of mRNA expression in oocytes showing changes in a subset of genes after *Rps26* deletion in *Rps26*^*fl/fl*^*/Zp3-Cre* (RZC) and *Rps26* ^*fl/fl*^*/Gdf9-Cre* (RGC) mice compared with the control (CTRL) *Rps26*^*fl/fl*^ mice at PD14 and PD28. **b** Gene ontology showing the biological processes affected by the altered mRNA expression by comparing the gene expression in the different groups of oocytes at PD14 (2w) and PD28 (4w).**c**–**d** Numbers of upregulated (up) genes and downregulated (down) genes in oocytes of control (CTRL) *Rps26*^*fl/fl*^ mice (**c**) and *Rps26* ^*fl/fl*^*/Gdf9-Cre* mice (**d**) at PD28 compared to PD14. **e**–**f** Numbers of upregulated (up) genes and downregulated (down) genes in oocytes of *Rps26* ^*fl/fl*^*/Gdf9-Cre* mice compared to *Rps26*^*fl/fl*^ mice at PD14 (**e**) and PD28 (**f**). **g** Genes of histone methyltransferases of H3K4 or H3K9 and other related genes were downregulated in *Rps26* ^*fl/fl*^*/Gdf9-Cre* mice compared to *Rps26*^*fl/fl*^ mice as determined from RNA sequencing. *p *<* 0.05 as calculated by two-tailed Student’s *t*-tests. Genes involved in DNA methylation and oocyte-specific genes were mostly downregulated in *Rps26* ^*fl/fl*^*/Gdf9-Cre* mice compared to *Rps26*^*fl/fl*^ mice as determined by RNA sequencing. *p *<* 0.05 as calculated by two-tailed Student’s *t*-tests

To determine the variation in gene expressions, we performed single-cell RNA sequencing of the oocytes. High-quality reads were filtered, leaving about 80% clean reads for analyses (Fig. S6A). Distribution of the global gene expression of each sample showed proximal values (Fig. S6B). A subset of mRNAs showed differential expressions in *Rps26*^*fl/fl*^*/Gdf9-Cre* or *Rps26*^*fl/fl*^*/Zp3-Cre* oocytes compared with *Rps26*^*fl/fl*^ oocytes from PD14 and PD28 mice (Fig. 5a). Gene ontology revealed that the discrepant genes were mainly involved in development, gene transcription and RNA synthesis (Fig. 5b). A total of 1590 genes were altered, 789 genes were upregulated and 801 were downregulated, with about 50% genes being downregulated in *Rps26*^*fl/fl*^ oocytes from PD28 mice compared to PD14 mice (Fig. 5c). However, there was only a total of 1128 genes were altered, 417 genes were upregulated and 711 genes were downregulated, 63.03% of the altered genes were downregulated in *Rps26*^*fl/fl*^*/Gdf9-Cre* oocytes from PD28 mice compared to PD14 mice. This suggests the unbalanced transcription in *Rps26*^*fl/fl*^*/Gdf9-Cre* oocytes compared to *Rps26*^*fl/fl*^ oocytes (Fig. 5d). At PD14, a total of 964 genes were altered, 515 genes were up-regulated and 449 genes were down-regulated in *Rps26*^*fl/fl*^*/Gdf9-Cre* oocytes compared to *Rps26*^*fl/fl*^ oocytes (Fig. 5e). While at PD28, there was a total of 4350 genes were altered, 2085 genes were upregulated, and 2265 genes were downregulated in *Rps26*^*fl/fl*^*/Gdf9-Cre* oocytes compared with *Rps26*^*fl/fl*^ oocytes (Fig. 5f). This indicated that gene expression in *Rps26*^*fl/fl*^*/Gdf9-Cre* oocytes at PD28 had been globally disrupted. Most genes of histone methyltransferases of H3K4 and H3K9 such as *Dnmt1*, *Setdb1*, *Cxxc1*, *Dnmt3a*, *Dnmt3b*, and other related genes in *Rps26*^*fl/fl*^*/Gdf9-Cre* oocytes were widely downregulated compared to *Rps26*^*fl/fl*^ oocytes in PD14 mice, and statistically significant differences were seen for *Dnmt1* and *Mllt10* (Fig. 5g). In addition, genes involved in DNA methylation, such as *Ddb1*, *Cnot7*, and *Tet3* were reduced on mRNA level, and the oocyte-specific genes *Oosp1*, *Zar1*, and *Rfpl4* were downregulated, and significant differences were seen for *Cnot7* and *Oosp1* in *Rps26*^*fl/fl*^*/Gdf9-Cre* oocytes compared to *Rps26*^*fl/fl*^ (Fig. 5h). This suggested that Rps26 regulates a subset of mRNAs that their proteins were important for epigenetic modifications. The relative expressions of other ribosomal genes were found mostly upregulated in *Rps26*^*fl/fl*^*/Gdf9-Cre* oocytes compared to *Rps26*^*fl/fl*^ oocytes at PD14 (Fig. S7C). This might be an attempt to compensate for the loss of *Rps26*.

### Downregulation of the PI3K/Akt/Foxo3a pathway might arrest oocyte growth and follicle development

**Fig. 6:**
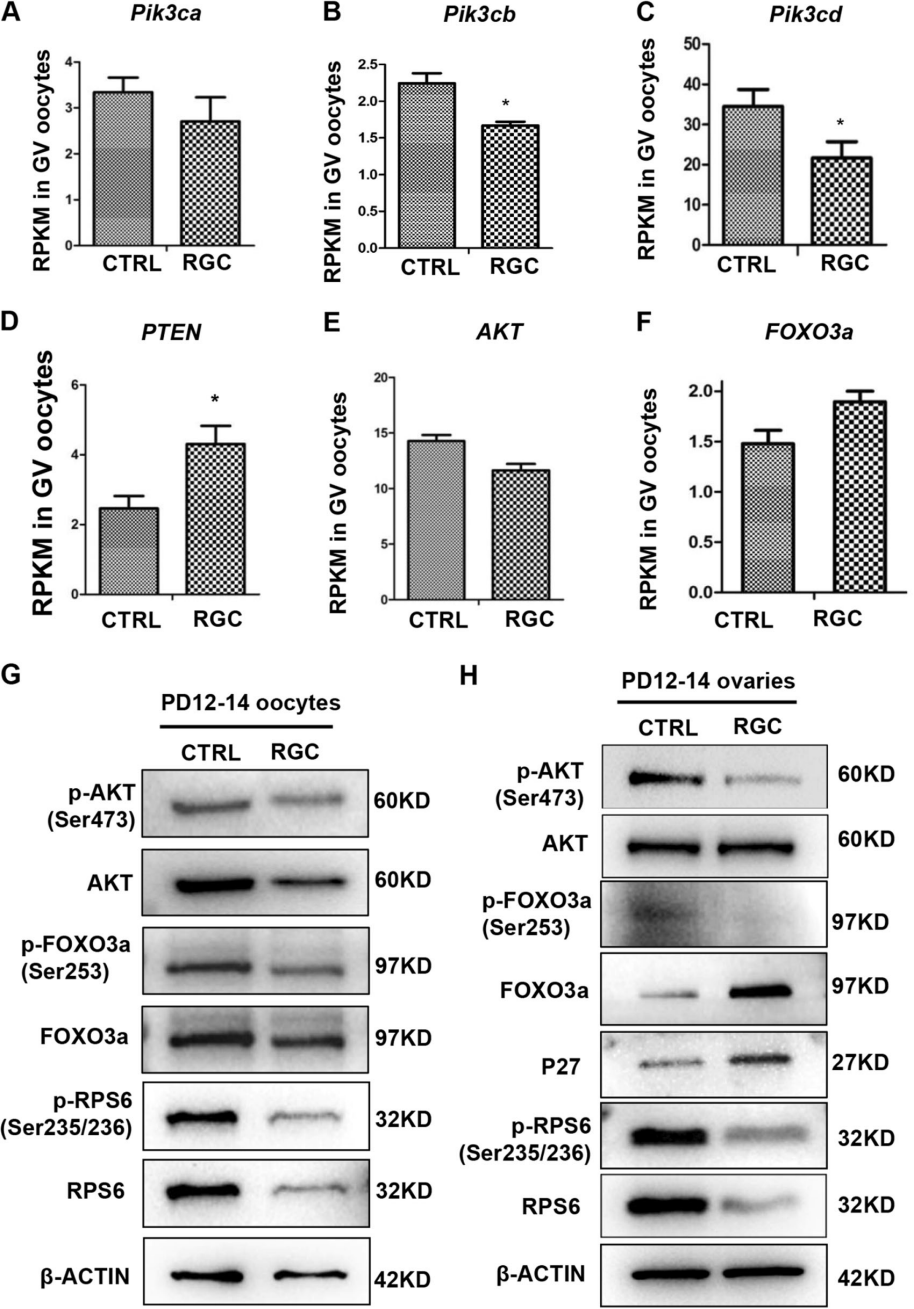
The PI3K/Akt/Foxo3a signaling pathway was down-regulated in *Rps26* knockout oocytes and ovaries. **a**–**c** The mRNA expressions of *Pi3k3ca* (**a**), *Pi3k3cb* (**b**), and *Pik3cd* (**c**) were decreased in oocytes from PD14 *Rps26*^*fl/fl*^*/Gdf9-Cre* (RGC) mice compared to control (CTRL) *Rps26*^*fl/fl*^ mice, as analyzed by RNA sequencing. *p *<* 0.05 as calculated by two-tailed Student’s *t*-tests. **d**, **f** The mRNA expressions of *Pten* (**d**) and *Foxo3a* (**f**) were increased in *Rps26*^*fl/fl*^*/Gdf9-Cre* (RGC) mice compared to control (CTRL) *Rps26*^*fl/fl*^ mice. *p *<* 0.05 as calculated by two-tailed Student’s *t*-tests. **e** The mRNA expression of *Akt* was decreased in oocytes of *Rps26*^*fl/fl*^*/Gdf9-Cre* (RGC) mice compared to control (CTRL) *Rps26*^*fl/fl*^ mice. **g** Western blot results showing the expression of p-Akt (Ser473), Akt, p-Foxo3a (Ser253), Foxo3a, p-Rps6 (Ser235/236), and Rps6 in the oocytes from *Rps26*^*fl/fl*^*/Gdf9-Cre* (RGC) mice and control (CTRL) *Rps26*^*fl/fl*^ mice at the age of PD12–14. **h** Western blot results showing reduced expression of the p-Akt (Ser473), p-Foxo3a (Ser253), p-Rps6 (Ser235/236), and Rps6 proteins and increased expression of the Foxo3a and P27 proteins in the PD12–14 ovaries of *Rps26*^*fl/fl*^*/Gdf9-Cre* (RGC) mice compared to control (CTRL) *Rps26*^*fl/fl*^ mice

Given the similarity in phenotypes of retarded oocyte growth and infertility in mice between constitutive expression of Foxo3a in oocytes and present *Rps26*^*fl/fl*^*/Gdf9-Cre* mice, we hypothesized that disruption of PI3K/Akt/Foxo3a pathway might be responsible for the failure of oocyte maturation and infertility of *Rps26*^*fl/fl*^*/Gdf9-Cre* females. The mRNA sequencing data showed that PI3K/Akt/Foxo3a pathway was significantly downregulated in *Rps26*^*fl/fl*^*/Gdf9-Cre* oocytes. The expression of the genes encoding the three main subunits of PI3K – *Pik3ca*, *Pik3cb*, and *Pik3cd*—was decreased (Fig. 6a, b, c), while *Pten* was increased (Fig. 6d). Moreover, the expression of *Akt* was reduced (Fig. 6e), and the *Foxo3a* was increased (Fig. 6f), while only *Pik3cb*, *Pik3cd*, and *Pten* showed significant differences. Protein levels of phosphorylation of Akt (Ser473) and total Akt were both reduced in oocytes from ovaries of *Rps26*^*fl/fl*^*/Gdf9-Cre* mice compared to *Rps26*^*fl/fl*^ mice (Fig. 6g). This suggested that downregulation of PI3K/Akt/Foxo3a pathway might contribute to the arrest of oocyte growth in *Rps26*^*fl/fl*^*/Gdf9-Cre* mice. In addition, ribosomal protein S6 (Rps6), a downstream factor of PI3K/Akt, is associated with mRNA translation and is known to mediate cell cycle. It was found that phosphorylated Rps6 (Ser235/236; p-Rps6) and total Rps6 were both maintained at low levels in oocytes of *Rps26*^*fl/fl*^*/Gdf9-Cre* mice compared to *Rps26*^*fl/fl*^ mice (Fig. 6g), suggesting that the mRNA translation involved in cell cycle was severely suppressed in oocytes.

In addition, phosphorylation of Akt, Foxo3a, and Rps6 proteins was suppressed in the ovaries of *Rps26*^*fl/fl*^*/Gdf9-Cre* mice compared to *Rps26*^*fl/fl*^ mice (Fig. 6h). While the downstream cell cycle inhibitor P27 was upregulated, Rps6 and p-Rps6 were both maintained at low levels in ovaries of *Rps26*^*fl/fl*^*/Gdf9-Cre* mice compared to *Rps26*^*fl/fl*^ mice, indicating that the translation of mRNAs involved in cell cycle was globally suppressed in ovaries of *Rps26*^*fl/fl*^*/Gdf9-Cre* mice (Fig. 6h). These results indicate that the downregulation of PI3K/Akt/Foxo3a pathway might lead to the arrest of oocyte growth and the retardation of follicle development in *Rps26*^*fl/fl*^*/Gdf9-Cre* mice.

### The reduction of oocyte-derived Gdf9, Bmp15, and Cx37 arrested ovary growth

**Fig. 7:**
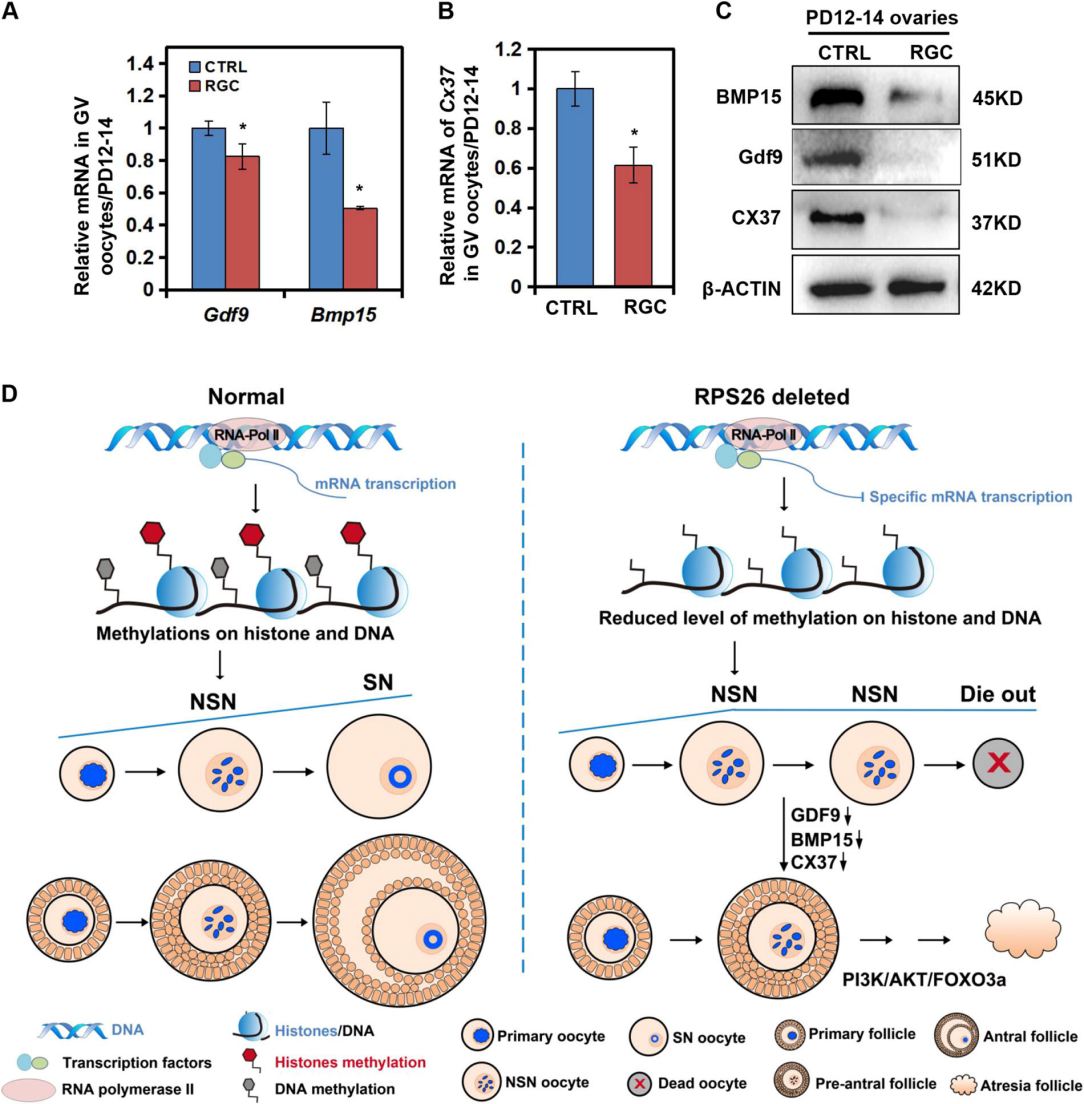
Downregulation of the oocyte-derived factors Bmp15, Gdf9, and Cx37 and the proposed working model. **a** The mRNA expression of *Gdf9* and *Bmp15* was decreased in oocytes of *Rps26*^*fl/fl*^*/Gdf9-Cre* (RGC) mice compared to control (CTRL) *Rps26*^*fl/fl*^ mice. *p *<* 0.05 as calculated by two-tailed Student’s *t*-tests. **b** The mRNA expression of *Cx37* was decreased in oocytes of *Rps26*^*fl/fl*^*/Gdf9-Cre* (RGC) mice compared to control (CTRL) *Rps26*^*fl/fl*^ mice. *p *<* 0.05 as calculated by two-tailed Student’s *t*-tests. **c** Western blot results showing reduced expression of the Bmp15, Gdf9, and Cx37 proteins in PD12–14 ovaries from *Rps26*^*fl/fl*^*/Gdf9-Cre* (RGC) mice compared with control (CTRL) *Rps26*^*fl/fl*^ mice. **d** The proposed working model. Generally, normal oocytes in primary follicles increase in mRNA synthesis activity mainly through RNA polymerase II, and they experience gradually increased levels of histone and DNA methylation, their nucleolus gradually transforms from the NSN to SN-type, and the follicles develop from pre-antral to antral follicles. Deletion of *Rps26* in oocytes leads to reduced mRNA synthesis activity through downregulation of RNA polymerase II, and this leads to reduced levels of methylation modifications of histones and DNA and subsequent failure to transition from the NSN to SN-type. The oocytes secrete significantly lower levels of Gdf9, Bmp15, and Cx37, and this prevents follicle development from pre-antral follicles to antral follicles. In addition, inhibition of the PI3K/Akt/Foxo3a pathway in the ovary arrests the growth of NSN-type oocytes, and the oocytes finally die along with follicle degeneration, which ultimately leads to POF

To determine the reason why the overall ovary growth was arrested in *Rps26*^*fl/fl*^*/Gdf9-Cre* mice, we measured the levels of two key paracrine growth factors Gdf9 and Bmp15, and the gap junction protein Cx37 in PD14 *Rps26*^*fl/fl*^*/Gdf9-Cre* and *Rps26*^*fl/fl*^ mice. The mRNA levels of *Gdf9* and *Bmp15* were significantly decreased in oocytes, and their protein levels in the ovaries were greatly decreased in *Rps26*^*fl/fl*^*/Gdf9-Cre* mice compared to *Rps26*^*fl/fl*^ mice (Fig. 7a, c). In addition, the mRNA and protein levels of Cx37 were significantly reduced in oocytes and ovaries of PD14 *Rps26*^*fl/fl*^*/Gdf9-Cre* mice (Fig. 7b, c). Insufficient expression of Gdf9 and Bmp15 in oocytes likely contributes to the arrest of oocytes growth in *Rps26*^*fl/fl*^*/Gdf9-Cre* mice, and the absence of Cx37 likely acts as a barrier in the communication between oocytes and granulosa cells leading to the loss of follicles and subsequent POF.

## Discussion

Rps26 was suggested to bind certain mRNAs^4^, and suppress its own pre-mRNA to regulate the translation^37^. The human ovary shows high mRNA expression of *RPS26* that is implicated in the susceptibility to PCOS^7^, suggestive of its important roles in human ovary. Since mouse and human RPS26 protein show 95.7% similarity (Fig. S1B), it is convenient to study the reproductive functions of Rps26 in mice. Thus, we constructed the oocyte-specific knockout of *Rps26* in mice to study its function (Fig. S1C).

Rps26 was expressed in both oocytes and granulosa cells of the mouse ovary, while *Rps26* deletion in oocytes led to arrest in oocytes growth and decrease in ovary size since PD14. As a consequence, *Rps26*^*fl/fl*^*/Gdf9-Cre* oocytes were arrested in NSN-type oocytes during PD21–28. These NSN-type oocytes showed a lack of histone and DNA methylation and were hard to complete meiosis process in *Rps26*^*fl/fl*^*/Gdf9-Cre* mice. These oocytes were arrested inside the pre-antral follicles and eventually died off, which led to POF (Fig. 7). Since *Rps26* was susceptibility to PCOS, and PCOS patients have higher risk for POF^38^. It seemed that our research mimics the process in which PCOS finally leads to POF.

Growing oocytes generally undergo NSN to SN transition at PD18 to form fully grown oocytes^14^. NSN oocytes in pre-antral follicles mostly show reduced rates of meiotic maturation in vitro^14,15,17^, which was confirmed in our study. Since the follicles in *Rps26*^*fl/fl*^*/Gdf9-Cre* ovary were mostly arrested at the pre-antral follicle stage, thus the small size of the ovary and the infertility are associated with the failure to undergo the NSN to SN transition. Epigenetic modifications are reported to be high in SN-type oocytes, while low in NSN-type oocytes^13,16^. And oocytes from the PD21 *Rps26*^*fl/fl*^*/Gdf9-Cre* mice showed low levels of H3K4me3, H3K9me3 and 5mC, all of which led to variations in gene expression. Although *Rps26* was knocked-out, the nucleolus structure, which is important for ribosomal RNA transcription and processing, was not disrupted in *Rps26*^*fl/fl*^*/Gdf9-Cre* oocytes (Fig. S6A, B), other ribosomal genes were increased on mRNA level (Fig. S6C), which might compensate for the loss of *Rps26*. This indicated that loss of *Rps26* led to variations on a subset of genes, and this might be associated with variations of epigenetic modifications. We hypothesize that the arrest in the NSN-type oocytes is associated with the low level of epigenetic modifications and the retardation in follicle development in *Rps26*^*fl/fl*^*/Gdf9-Cre* mice.

Granulosa cells regulate the transcriptional activity of the oocytes, highlighting the importance of gap junctions between oocytes and granulosa cells^27,31,39^. Cx37 was greatly decreased in *Rps26*^*fl/fl*^*/Gdf9-Cre* ovaries, which prevented the proliferation or differentiation of granulosa cells from the regulation of oocytes. In addition, the reduced Bmp15 and Gdf9 further inhibited oocyte and follicle growth in *Rps26*^*fl/fl*^*/Gdf9-Cre* ovaries.

Follicle activation and development are regulated by the PI3K/Akt/Foxo3a pathway^22^, constitutive activation of Foxo3a in oocytes causes retardation in oocyte growth and follicle development^26^. Our study further demonstrated that increased expressions of Foxo3a and P27 contributed to retardation of follicle development in *Rps26*^*fl/fl*^*/Gdf9-Cre* mice. In addition, p-Akt and p-Rps6 were reduced in both oocytes and ovaries, contributing to the inhibition of oocyte growth and follicle development.

To sum up, our study reveals a novel function for Rps26 in the regulation of oocyte chromatin configurations, and that Rps26 maintains oocyte growth and follicle development in female mouse. Thus, our study might provide genetic clues for the prediction of POF.

## Materials and methods

### Animals

The *Rps26*^*fl/fl*^ mice in a C57BL/6J genomic background were obtained from Cyagen Biosciences, China. *Gdf9-Cre* and *Zp3-Cre* mice were derived as previously reported and crossed in an ICR genomic background^40^. *Rps26*^*fl/fl*^ mice were crossed with *Gdf9-Cre* and *Zp3-Cre* mice to produce the oocyte-specific *Rps26* knockout in *Rps26*^*fl/fl*^*/Gdf9-Cre* and *Rps26*^*fl/fl*^*/Zp3-Cre* mice in a mixed genomic background. Mice were bred under specific pathogen-free conditions with a 12-h light–dark cycle and free access to water and food. This research was in compliance with the ethical legislation for animal research.

### Reagent and antibodies

Human chorionic gonadotropin (hCG) and PMSG were purchased from Ningbo Sansheng Pharmaceutical Co. (China). M16 media and hyaluronidase were obtained from Sigma-Aldrich (Germany). The anti-fade reagent with DAPI (4′,6-diamidino-2-phenylindole, dihydrochloride) was supplied by Life Technologies Corp. (Carlsbad, CA, USA).

The antibody against Rps26 was obtained from Proteintech Group, Inc., while antibodies against H3K4me3, H3K9me3, RNA polymerase II, p-RNA polymerase II (p-S2), Gdf9, Bmp15, and Cx37 were purchased from Abcam. Antibodies against p-Akt (Ser473), Akt, p-Rps6 (Ser235/236), Rps6, p-Foxo3a (Ser253), Foxo3a, and actin were purchased from Cell Signaling Technology (Beverly, MA, USA). The antibody against P27 was purchased from Santa Cruz Biotechnology Inc., and the antibody against 5mC was purchased from Calbiochem Co. The Click-iT® RNA Alexa Fluor® 594 imaging kit was obtained from Invitrogen Co. Ltd.

### In vitro oocyte meiosis and superovulation

Oocytes were collected from the ovaries of PD21–28 mice that were not treated with hCG and then transferred to M16 medium. These oocytes were cultured in M16 medium for 4 h or 12 h in vitro at 37 °C under 5% CO_2_. The extent of meiosis in the oocytes was determined based on the ratio of GVBD and polar body extrusion (PBE). For the superovulation assay, mice were injected with 5 IU PMSG, cultured for 44 h, and then injected with 5 IU hCG. After 16 h, the cumulus-oocyte complexes were disassociated from the oviducts and digested with hyaluronidase, and the number of oocytes was counted.

### Immunofluorescent microscopy for oocytes

Oocytes were fixed in 4% paraformaldehyde buffered with phosphate-buffered saline (PBS) for about 30 min at room temperature, followed by washing in PBST (0.1% Tween and 0.01% Triton X-100 in PBS). The oocytes were permeabilized in 0.2% Triton X-100 in PBS for 10 min, blocked in 1% bovine serum albumin for 30 min, and incubated with the primary antibody for 1–2 h at room temperature. Following incubation, the oocytes were washed three times with PBST and then incubated with secondary antibody for 30 min. The oocytes were mounted on slides in an anti-fade reagent with DAPI, and images were captured under a confocal laser-scanning microscope (Zeiss LSM 780, Carl Zeiss AG, Germany).

### Histological and morphological analysis

Mouse ovaries from different ages and treatment groups were fixed in 10% formalin overnight at 4 °C, dehydrated through a series of graded ethanol solutions and xylene, and embedded in paraffin. The ovaries were sectioned at a thickness of 5 μm, stained with hematoxylin and eosin reagents after deparaffinization, and imaged under an optical microscope.

### Click-iT assay

The 5-EU was added to the M16 medium and incubated with GV oocytes for 1 h. Following treatment, the oocytes were fixed in 4% paraformaldehyde in PBS for 30 min, permeabilized with 0.5% Triton X-100 in PBS for 15 min at room temperature, and incubated with the Click-iT reaction cocktail for 30 min at room temperature in the dark. After incubation, the oocytes were washed once with the Click-iT reaction rinse buffer, stained with Hoechst33342 for 15 min in the dark, and mounted on slides in the presence of anti-fade reagent. Images were captured under a confocal laser-scanning microscope (Zeiss LSM 780, Carl Zeiss AG, Germany).

### RNA sequencing

Every ten oocytes were set as one sample. RNAs were extracted using the RNeasy Mini Kit (Qiagen, Germany) and sequenced using the PE100 strategy (HiSeq2500, Illumina). The raw data were filtered to get high-quality clean data for analysis, and the expression levels of the mapped genes were evaluated by reads per kilo-base of exon per million mapped reads (RPKM). The sequencing was performed and analyzed using the Annoroad Gene Technology. Expression levels of mRNAs were partially verified through the quantified real-time PCR experiments on a Light Cycler® 480 (Roche, Germany).

### Western blot analysis

Every 200 oocytes from each genotype were collected in a sodium dodecyl sulfate (SDS) lysis buffer as one sample for each lane, heated for 5 min at 95 °C, and subjected to SDS polyacrylamide gel electrophoresis. The separated proteins were transferred onto polyvinylidene fluoride membranes and incubated overnight with the appropriate primary antibody at 4 °C. The membranes were then incubated with the appropriate secondary antibody for 1 h, and the protein bands were detected using the Bio-Rad gel imaging system.

### Statistical analysis

All results are shown as the mean ± standard deviation, each experiment was conducted independently at least three times with three replicates each. Group comparisons were made by two-tailed unpaired Student’s *t*-tests. A *p*-value < 0.05 was considered significant.

## Electronic supplementary material

Supplementary information

## References

[1] Yadaiah M (2013). Arrested cell proliferation through cysteine protease activity of eukaryotic ribosomal protein S4. FASEB J.: Off. Publ. Fed. Am. Soc. Exp. Biol..

[2] Wang W (2015). Ribosomal proteins and human diseases: pathogenesis, molecular mechanisms, and therapeutic implications. Med. Res. Rev..

[3] Brogna S, Sato TA, Rosbash M (2002). Ribosome components are associated with sites of transcription. Mol. Cell.

[4] Sharifulin D (2012). A central fragment of ribosomal protein S26 containing the eukaryote-specific motif YxxPKxYxK is a key component of the ribosomal binding site of mRNA region 5’ of the E site codon. Nucl. Acids Res..

[5] Rabl J, Leibundgut M, Ataide SF, Haag A, Ban N (2011). Crystal structure of the eukaryotic 40S ribosomal subunit in complex with initiation factor 1. Sci. (New Y., N. Y.).

[6] Fagerberg L (2014). Analysis of the human tissue-specific expression by genome-wide integration of transcriptomics and antibody-based proteomics. Mol. Cell. Proteom.: MCP.

[7] Shi Y (2012). Genome-wide association study identifies eight new risk loci for polycystic ovary syndrome. Nat. Genet..

[8] Labrecque R, Sirard MA (2014). The study of mammalian oocyte competence by transcriptome analysis: progress and challenges. Mol. Hum. Reprod..

[9] Gao F (2014). Wt1 functions in ovarian follicle development by regulating granulosa cell differentiation. Hum. Mol. Genet..

[10] Fan HY (2009). MAPK3/1 (ERK1/2) in ovarian granulosa cells are essential for female fertility. Sci. (New Y., N. Y.).

[11] Eppig JJ, Wigglesworth K, Pendola FL (2002). The mammalian oocyte orchestrates the rate of ovarian follicular development. Proc. Natl Acad. Sci. U.S.A..

[12] Matzuk MM, Burns KH, Viveiros MM, Eppig JJ (2002). Intercellular communication in the mammalian ovary: oocytes carry the conversation. Sci. (New Y., N. Y.).

[13] Kageyama S (2007). Alterations in epigenetic modifications during oocyte growth in mice. Reproduction.

[14] Wickramasinghe D, Ebert KM, Albertini DF (1991). Meiotic competence acquisition is associated with the appearance of M-phase characteristics in growing mouse oocytes. Dev. Biol..

[15] Mattson BA, Albertini DF (1990). Oogenesis: chromatin and microtubule dynamics during meiotic prophase. Mol. Reprod. Dev..

[16] Bao S, Obata Y, Carroll J, Domeki I, Kono T (2000). Epigenetic modifications necessary for normal development are established during oocyte growth in mice. Biol. Reprod..

[17] Bouniol-Baly C (1999). Differential transcriptional activity associated with chromatin configuration in fully grown mouse germinal vesicle oocytes. Biol. Reprod..

[18] Fischle W, Wang Y, Allis CD (2003). Histone and chromatin cross-talk. Curr. Opin. Cell Biol..

[19] Nakayama J, Rice JC, Strahl BD, Allis CD, Grewal SI (2001). Role of histone H3 lysine 9 methylation in epigenetic control of heterochromatin assembly. Sci. (New Y., N. Y.).

[20] Matsumura Y (2015). H3K4/H3K9me3 bivalent chromatin domains targeted by lineage-specific DNA methylation pauses adipocyte differentiation. Mol. Cell.

[21] Bao RM, Hayakawa K, Moniruzzaman M, Taketsuru H, Miyano T (2011). FOXO3 knockdown accelerates development of bovine primordial follicles. J. Reprod. Dev..

[22] Li J (2010). Activation of dormant ovarian follicles to generate mature eggs. Proc. Natl. Acad. Sci. U.S.A..

[23] John GB, Gallardo TD, Shirley LJ, Castrillon DH (2008). Foxo3 is a PI3K-dependent molecular switch controlling the initiation of oocyte growth. Dev. Biol..

[24] Brunet A (1999). Akt promotes cell survival by phosphorylating and inhibiting a Forkhead transcription factor. Cell.

[25] Reddy P (2005). Activation of Akt (PKB) and suppression of FKHRL1 in mouse and rat oocytes by stem cell factor during follicular activation and development. Dev. Biol..

[26] Pelosi E (2013). Constitutively active Foxo3 in oocytes preserves ovarian reserve in mice. Nat. Commun..

[27] Liu L (2007). Infertility caused by retardation of follicular development in mice with oocyte-specific expression of Foxo3a. Development.

[28] Persani L, Rossetti R, Di Pasquale E, Cacciatore C, Fabre S (2014). The fundamental role of bone morphogenetic protein 15 in ovarian function and its involvement in female fertility disorders. Hum. Reprod. Update.

[29] Elvin JA, Yan C, Matzuk MM (2000). Growth differentiation factor-9 stimulates progesterone synthesis in granulosa cells via a prostaglandin E2/EP2 receptor pathway. Proc. Natl Acad. Sci. U.S.A..

[30] Mao GK (2013). Gap junction -mediated cAMP movement between oocytes and somatic cells. Front. Biosci. (Elite Ed.).

[31] Shuhaibar LC (2015). Intercellular signaling via cyclic GMP diffusion through gap junctions restarts meiosis in mouse ovarian follicles. Proc. Natl Acad. Sci. U.S.A..

[32] Simon AM, Goodenough DA, Li E, Paul DL (1997). Female infertility in mice lacking connexin 37. Nature.

[33] Kidder GM, Mhawi AA (2002). Gap junctions and ovarian folliculogenesis. Reproduction.

[34] McGrath SA, Esquela AF, Lee SJ (1995). Oocyte-specific expression of growth/differentiation factor-9. Mol. Endocrinol. (Baltim., Md.).

[35] Lewandoski M, Wassarman KM, Martin GR (1997). Zp3-cre, a transgenic mouse line for the activation or inactivation of loxP-flanked target genes specifically in the female germ line. Curr. Biol.: CB.

[36] Sun QY, Liu K, Kikuchi K (2008). Oocyte-specific knockout: a novel in vivo approach for studying gene functions during folliculogenesis, oocyte maturation, fertilization, and embryogenesis. Biol. Reprod..

[37] Ivanov AV, Malygin AA, Karpova GG (2005). Human ribosomal protein S26 suppresses the splicing of its pre-mRNA. Biochim. Biophys. Acta.

[38] Pan ML, Chen LR, Tsao HM, Chen KH (2017). Polycystic ovarian syndrome and the risk of subsequent primary ovarian insufficiency: a nationwide population-based study. Menopause (New Y., N. Y.).

[39] De La Fuente R, Eppig JJ (2001). Transcriptional activity of the mouse oocyte genome: companion granulosa cells modulate transcription and chromatin remodeling. Dev. Biol..

[40] Lan ZJ, Xu X, Cooney AJ (2004). Differential oocyte-specific expression of Cre recombinase activity in GDF-9-iCre, Zp3cre, and Msx2Cre transgenic mice. Biol. Reprod..

