# Supplementary material for: Loss of oocyte Rps26 in mice arrests oocyte growth and causes premature ovarian failure
Source: Cell Death Dis. 2018 Nov 19;9(12):1144. doi: 10.1038/s41419-018-1196-3 (PMC6242890; doi:10.1038/s41419-018-1196-3)
Supplement: Supplementary file 1 — Supplementary information [file 41419_2018_1196_MOESM1_ESM.docx]

Supplementary Information

**Loss of oocyte Rps26 in mice arrests oocyte growth and causes premature ovarian failure**

Xiao-Man Liu,^1,2,3^ Ming-Qi Yan,^1,2,3^ Shu-Yan Ji,^6^ Qian-Qian Sha,^6^ Tao Huang,^1,2,3^ Han Zhao,^1,2,3^ Hong-Bin Liu, ^1,2,3^ Heng-Yu Fan,^6^ and Zi-Jiang Chen^1,2,3,4,5^

Email: [chenzijiang@hotmail.com](mailto:chenzijiang@hotmail.com)

**This PDF file includes:**

Figs. S1 to S7

Tables S1 to S2

Figure S1


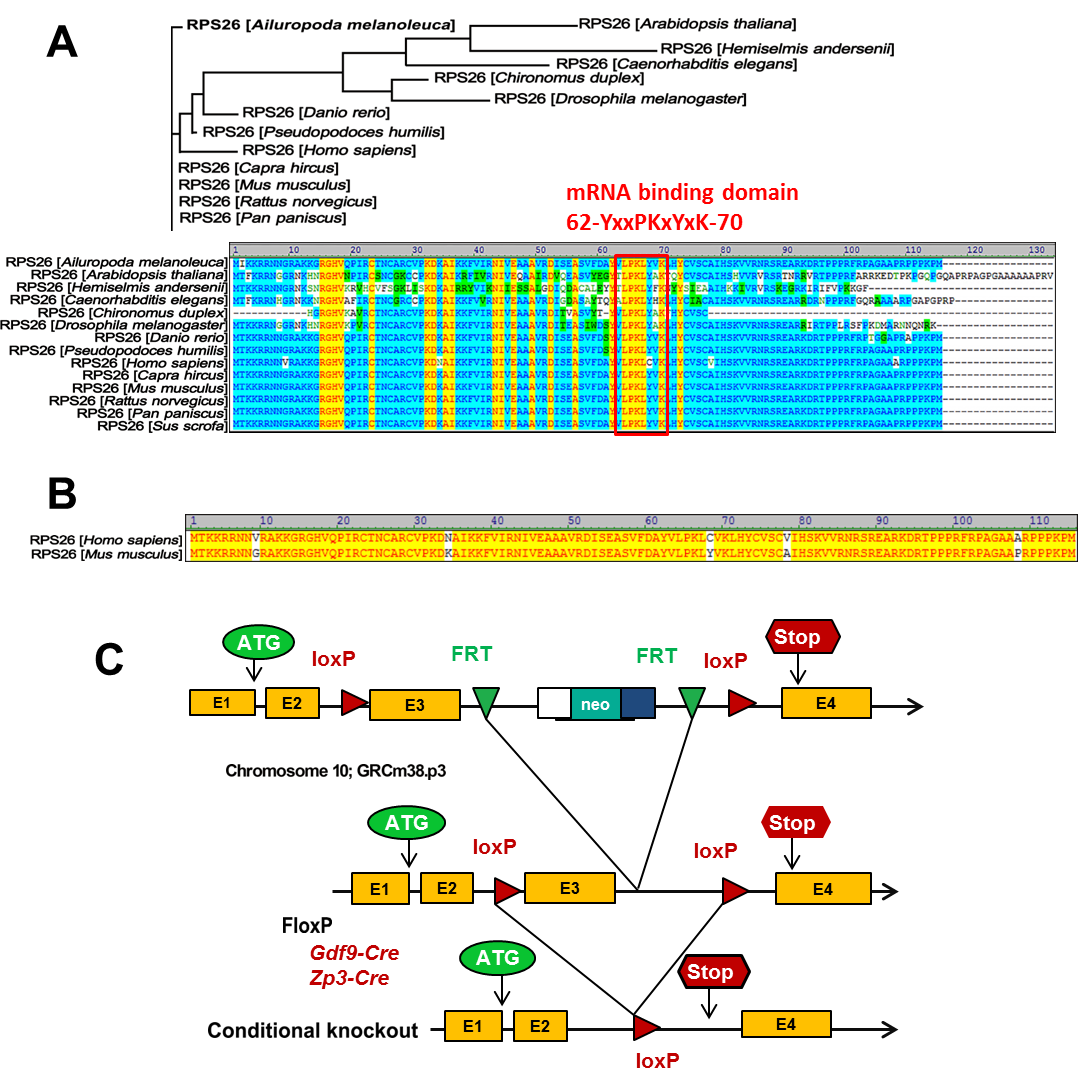


**Fig. S1. RPS26 is a highly conserved protein in the evolution, and the conditional knockout strategy targeting *Rps26* gene in mouse. Related to Figure 1.**

(A) Alignment of RPS26 protein amino acid sequences from *Hemiselmis andersenii* to *Homo sapiens,* which shows a sequence similarity of 86.5% and a potential mRNA binding domain. (B) Alignment of the human and mouse RPS26 protein amino acid sequence, which shows 95.7% similarity. (C) The knockout strategy for *Rps26^fl/fl^/Gdf9-Cre* and *Rps26^fl/fl^/Zp3-Cre* mice.

Figure S2


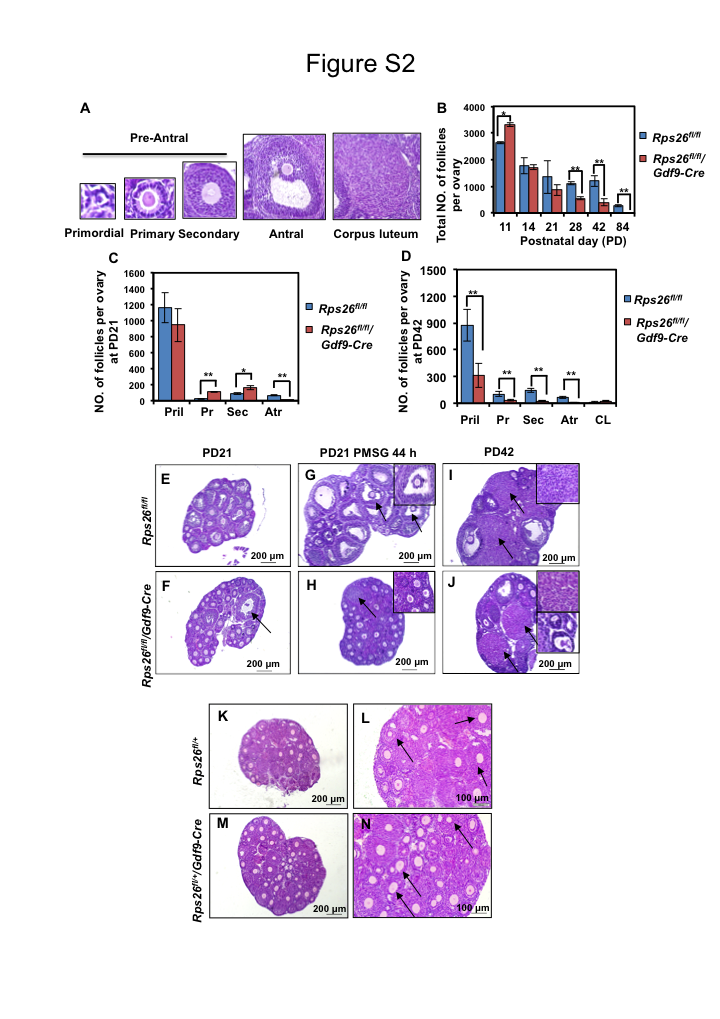


**Fig. S2. Follicle development was arrested at pre-antral follicles, but a few follicles could still develop and ovulate to form corpora lutea. Related to Figure 2.**

(A) The representative images of the follicles of primordial follicles, primary follicles, secondary folllciles, antral follicles and corpus luteum. (B) The total numbers of follicles in mice of *Rps26fl/fl* and *Rps26fl/fl/Gdf9-Cre* in different ages. (C) The numbers of primordial follicles (Pril), primary follicles (Pr), and secondary follicles (Sec) in indicated genotype mice at the age of PD21. (D) The numbers of primordial follicles (Pril), primary follicles (Pr), and secondary follicles (Sec) in indicated genotype mice at the age of PD42. (E-F) HE staining of PD21 mouse ovaries for the indicated genotypes *Rps26fl/fl* (E) and *Rps26fl/fl/Gdf9-Cre* in which one or two antral follicles are shown (F, arrow). (G-H) HE staining of ovaries of PD21 mice administered pregnant mare serum gonadotropin (PMSG) for the indicated genotypes *Rps26fl/fl* (G) and *Rps26fl/fl/Gdf9-Cre* (H). (I-J) HE staining of ovaries of PD42 mice for the indicated genotypes *Rps26fl/fl* (I) and *Rps26fl/fl/Gdf9-Cre* in which there were a few corpora lutea (J, arrows). (K, M) HE staining for ovaries of PD21 mice for the genotypes of *Rps26fl/+* (K) and *Rps26fl/+/Gdf9-Cre* (M). The enlarged images of *Rps26fl/+* (L) and *Rps26fl/+/Gdf9-Cre* (N) showing that several antral follicles could be found in both genotypes (L, N, arrows).

Figure S3


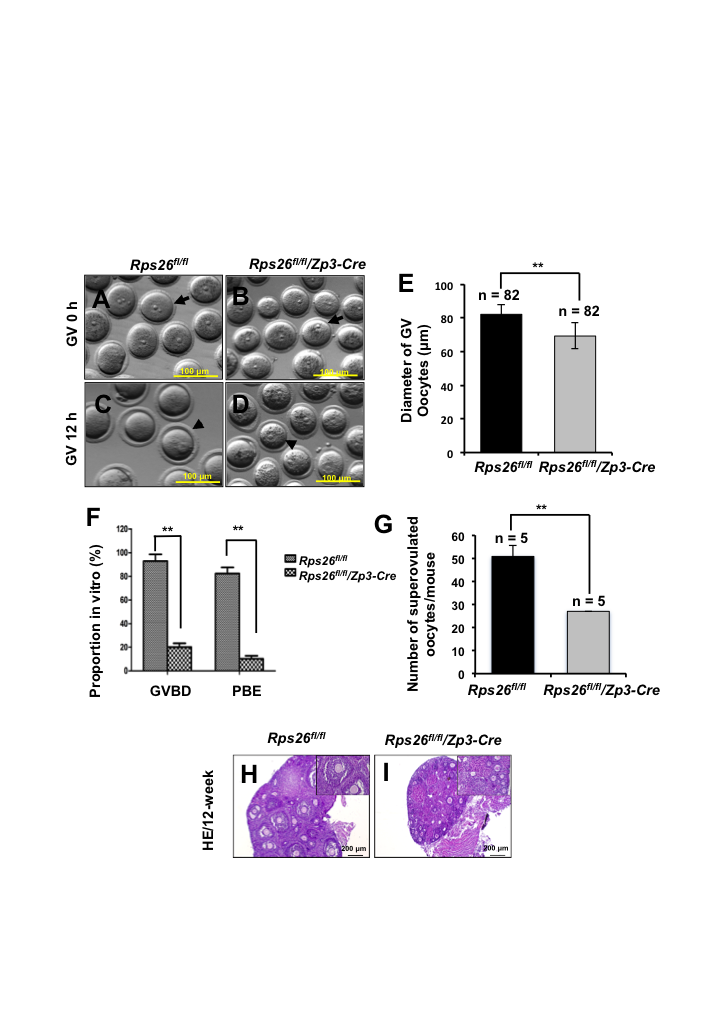


**Figure S3. Deletion of *Rps26* by Zp3-Cre arrested meiotic maturation and follicle development in oocytes. Related to Figure 2.**

1. B) GV oocytes of *Rps26^fl/fl^* mice (A) were larger in diameter than those from the ovaries of *Rps26^fl/fl^/Zp3-Cre* mice (B). (C-D) Oocyte meiosis was arrested at the GV stage in *Rps26^fl/fl^/Zp3-Cre* oocytes (D) after 12 h *in vitro* culture, while the oocytes of *Rps26^fl/fl^* mice developed into MII oocytes (C). (E) Diameters of GV oocytes from the ovaries of *Rps26^fl/fl^/ZP3-Cre* mice were significantly smaller than the diameters of oocytes from the ovaries of *Rps26^fl/fl^* mice. (F) The proportions of GVBD and PBE were significantly decreased in the oocytes from the ovaries of *Rps26^fl/fl^/Zp3-Cre* mice *in vitro* as compared with *Rps26^fl/fl^* mouse oocytes. (G) Oocyte superovulation was inhibited in *Rps26^fl/fl^/ZP3-Cre* mice as compared with *Rps26^fl/fl^* mice. (H-I) HE staining of mouse ovaries at –PD84 for the genotypes *Rps26^fl/fl^* (H) and *Rps26^fl/fl^/Zp3-Cre* (I).

Figure S4


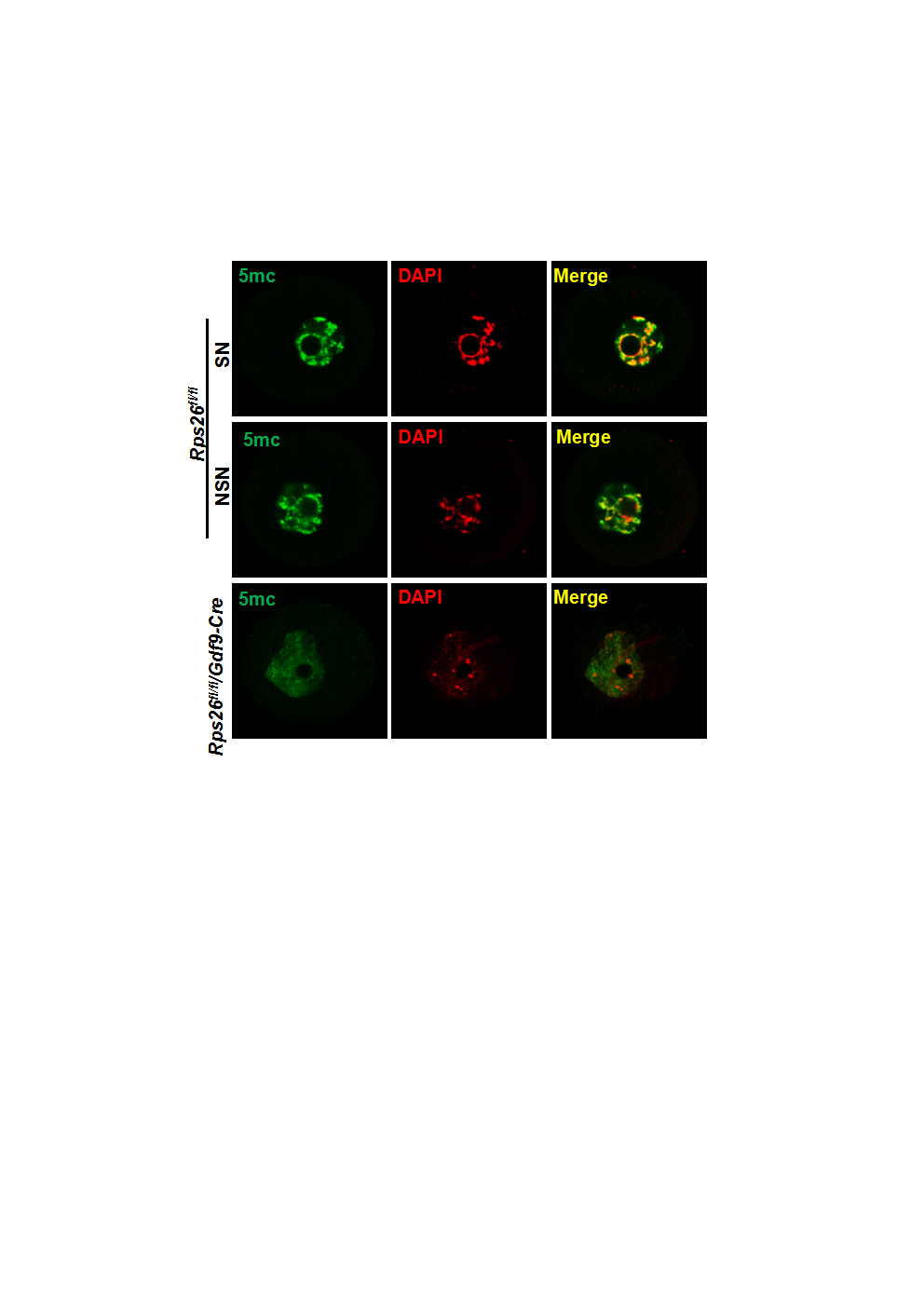


**Figure S4. Deletion of *Rps26* in GV oocytes led the failure of 5-methylation of cysteine (5mC) in the nucleus. Related to Figure 3.** There was a strong signal for 5mC in SN-type and NSN-type oocytes of PD21 *Rps26^fl/fl^* mice, but almost no signal in the oocytes of *Rps26^fl/fl^/Gdf9-Cre* mice in which most were NSN-type oocytes.

**Figure S5**


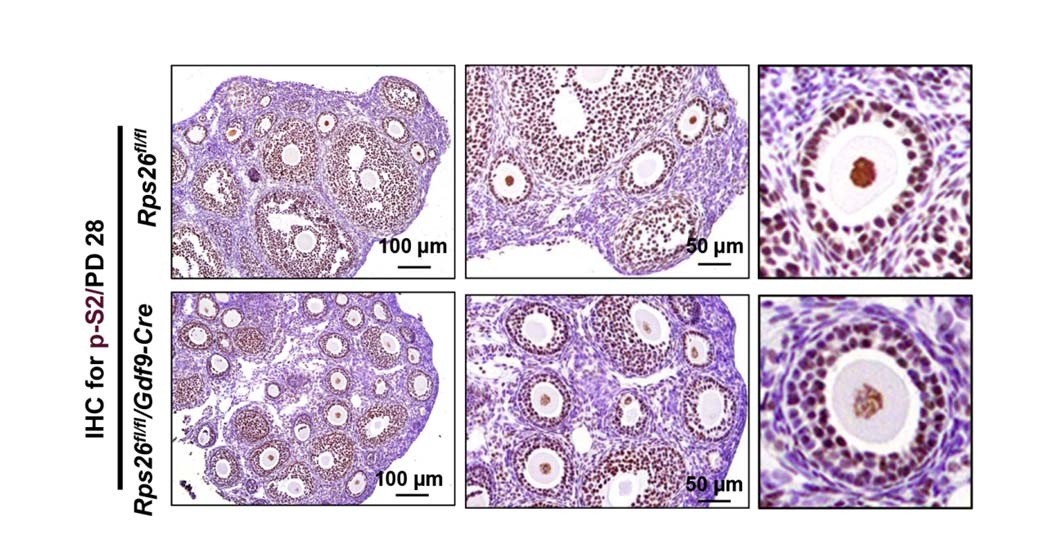


**Figure S5. Deletion of *Rps26* in GV oocytes led to low level of phosphorylation of RNA pol II in the nucleus. Related to Figure 4.** There was a low signal of the phosphorylation of RNA pol II (p-S2) in nucleus of oocytes of PD28 *Rps26fl/fl/Gdf9-Cre* mice, compared with the oocytes from *Rps26fl/f*  mice.

**Figure S6**

**
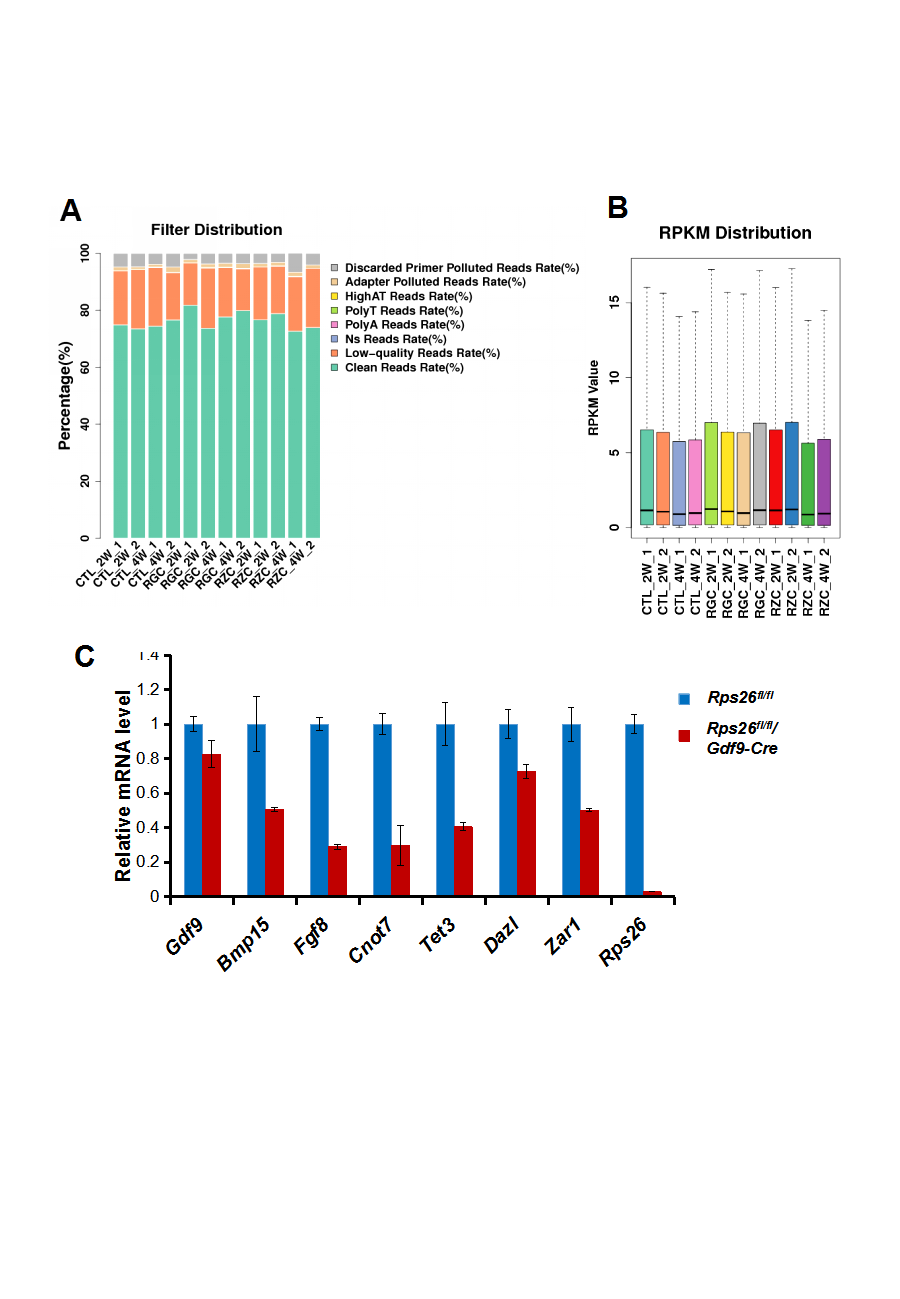
**

**Figure S6. Quality control and distribution of the single-cell transcription sequencing. Related to Figure 5.**

(A) Filter distribution in each sample, the clean reads were filtered for further analyses. (B) Distribution of the global gene expression in each samples.

(C) Several genes were selected and verified on mRNA level through RT-PCR.

**Figure S7**

**
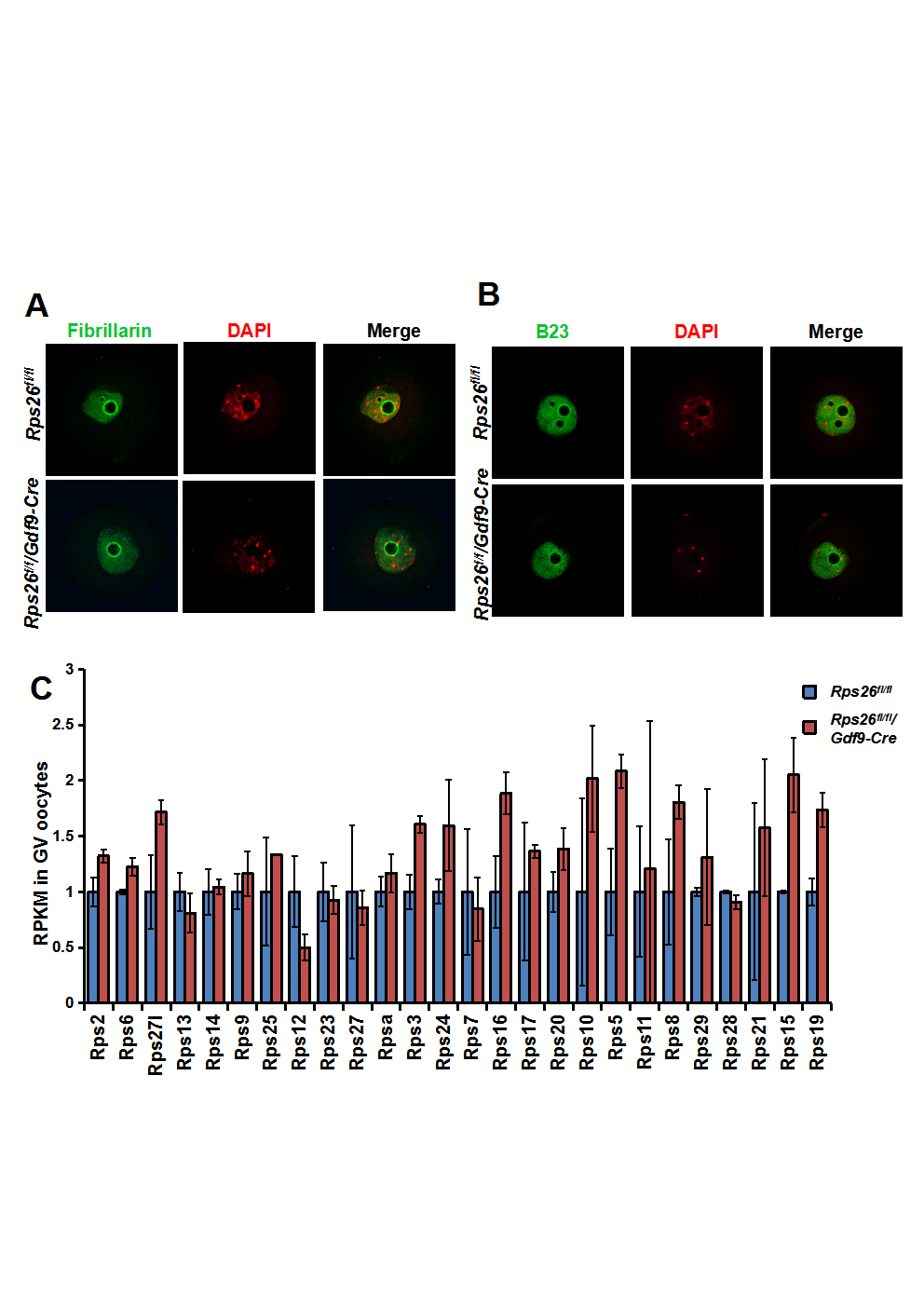
Figure S6. The structure of the nucleolus was normal in *Rps26^fl/fl^*/*Gdf9-Cre* oocytes.**

(A) The fibrillar center (FC) and dense fibrillar component (DFC) of the nucleolus as determined by immunofluorescence of fibrillarin showed normal morphology in *Rps26^fl/fl^*/*Gdf9-Cre* oocytes. (B) The granulosa component (GC) of the nucleolus as indicated by immunofluorescence of B23 showed normal morphology in *Rps26^fl/fl^/Gdf9-Cre* oocytes.

(C) Expression levels of ribosomal genes were generally increased in *Rps26^fl/fl^/Gdf9-Cre* oocytes at PD14 compared with *Rps26^fl/fl^* mice.

Table S1. Antibodies information.

| **Name of Antibody** | **Manufacturer (catalog number)** | **Dilution used** |
| --- | --- | --- |
| RPS26 | Proteintech (14909-1-AP) | WB (1:200); IF (1:200) |
| MVH | Abcam (104770) | IHC (1:500) |
| H3K4me3 | Abcam (8580) | IF (1:1000) |
| H3K9me3 | Abcam (8898) | IF (1:1000) |
| RNA polymerase II CTD repeat YSPTSPS (phospho S2) /pS2 | Abcam (70324) | IF (1:1000); WB (1:1000) |
| RNA polymerase II CTD repeat YSPTSPS | Abcam (52202) | IF (1:1000); WB (1:1000) |
| p-AKT(S473) | Cell Signaling ( 3787) | WB (1:500) |
| AKT | Cell Signaling (9272) | WB (1:500) |
| p-RPS6(Ser235/236) | Cell Signaling (2211) | WB (1:500) |
| RPS6 | Cell Signaling (2317) | WB (1:500) |
| β-Actin | Cell Signaling (4970) | WB (1:1000) |
| p-FOXO3a(S253) | Cell Signaling (13129) | WB (1:200) |
| FOXO3a | Cell Signaling (12829) | WB (1:200) |
| P27 | Santa Cruz (sc-528) | WB (1:50) |
| BMP15 | Abcam (108413) | WB (1:200) |
| GDF9 | Abcam (193443) | WB (1:200) |
| CX37 | Abcam (181701) | WB(1:500) |
| 5mC | Calbiochem (NA81) | IF (1:200) |
| B23 | Abcam (10530) | IF (1:1000) |
| Fibrillarin | Abcam (154806) | IF (1:1000) |

**Table S2. Primer sequences.**

| **Gene name** | **Sequence(5'-3')** | **Application** |
| --- | --- | --- |
| *Rps26* | 5'-GGTGCTGGGTCCTCAACTTA-3' | Genotyping |
|  | 5'-GCTGCGGTTGCCTACTTAAC-3' | Genotyping |
| *Gdf9-Cre* | 5'-GGTTTCTGTTGGGCTCTCAC-3' | Genotyping |
|  | 5'-ATCAGAGGTGGCATCCACAG-3' | Genotyping |
| *Zp3-Cre* | 5'-AAGAACCTGATGGACATGTTCAG-3' | Genotyping |
|  | 5'-CTGATCCTGGCAATTTCGG-3' | Genotyping |
| *Rps26* | F:CCAAGGATAAGGCCATCAAG | RT-PCR |
|  | R:AGAGCTTGGGAAGCACGTAG | RT-PCR |
| *Gapdh* | F:ACACTGAGGACCAGGTTGTCTC | RT-PCR |
|  | R:TACTCCTTGGAGGCCATGTAG | RT-PCR |
